# Effect of Mailing Educational Material to Patients With Atrial Fibrillation and Their Clinicians on Use of Oral Anticoagulants: A Randomized Clinical Trial

**DOI:** 10.1001/jamanetworkopen.2022.14321

**Published:** 2022-05-31

**Authors:** Sean D. Pokorney, Noelle Cocoros, Hussein R. Al-Khalidi, Kevin Haynes, Shuang Li, Sana M. Al-Khatib, Jacqueline Corrigan-Curay, Meighan Rogers Driscoll, Crystal Garcia, Sara B. Calvert, Thomas Harkins, Robert Jin, Daniel Knecht, Mark Levenson, Nancy D. Lin, David Martin, Debbe McCall, Cheryl McMahill-Walraven, Vinit Nair, Lauren Parlett, Andrew Petrone, Robert Temple, Rongmei Zhang, Yunping Zhou, Richard Platt, Christopher B. Granger

**Affiliations:** 1 Duke Clinical Research Institute and Division of Cardiology, Duke University Medical Center, Durham, North Carolina; 2 Harvard Pilgrim Health Care Institute, Harvard Medical School Department of Population Medicine, Boston, Massachusetts; 3 Duke Clinical Research Institute and Department of Biostatistics and Bioinformatics, Duke University Medical Center, Durham, North Carolina; 4 HealthCore Inc, Alexandria, Virginia; 5 Center for Drug Evaluation and Research, US Food and Drug Administration, Silver Spring, Maryland; 6 Clinical Trials Transformation Initiative, Durham, North Carolina; 7 Humana Healthcare Research Inc, Louisville, Kentucky; 8 Aetna Inc, Blue Bell, Pennsylvania; 9 Office of Biostatistics, Center for Drug Evaluation and Research, US Food and Drug Administration; 10 OptumInsight Life Sciences Inc, Boston, Massachusetts; 11 Moderna Inc, Cambridge, Massachusetts; 12 Rowan Tree Perspectives Consulting, Murrieta, California

## Abstract

**Importance:**

Only about half of patients with atrial fibrillation (AF) who are at increased risk for stroke are treated with an oral anticoagulant (OAC), despite guideline recommendations for their use. Educating patients with AF about prevention of stroke with OACs may enable them as agents of change to initiate OAC treatment.

**Objective:**

To determine whether an educational intervention directed to patients and their clinicians stimulates the use of OACs in patients with AF who are not receiving OACs.

**Design, Setting, and Participants:**

The Implementation of a Randomized Controlled Trial to Improve Treatment With Oral Anticoagulants in Patients With Atrial Fibrillation (IMPACT-AFib) trial was a prospective, multicenter, open-label, pragmatic randomized clinical trial conducted from September 25, 2017, to May 1, 2019, embedded in health plans that participate in the US Food and Drug Administration’s Sentinel System. It used the distributed database comprising health plan members to identify eligible patients, their clinicians, and outcomes. IMPACT-AFib enrolled patients with AF, a CHA_2_DS_2_-VASc (cardiac failure or dysfunction, hypertension, age 65-74 [1 point] or ≥75 years [2 points], diabetes, and stroke, transient ischemic attack or thromboembolism [2 points]–vascular disease, and sex category [female]) score of 2 or more, no evidence of OAC prescription dispensing in the preceding 12 months, and no hospitalization-related bleeding event within the prior 6 months.

**Interventions:**

Randomization to a single mailing of patient and/or clinician educational materials vs control.

**Main Outcomes and Measures:**

Analysis was performed on a modified intention-to-treat basis. The primary end point was the proportion of patients with at least 1 OAC prescription dispensed or at least 4 international normalized ratio test results within 1 year of the intervention.

**Results:**

Among 47 333 patients, there were 24 909 men (52.6%), the mean (SD) age was 77.9 (9.7) years, mean (SD) CHA_2_DS_2_-VASc score was 4.5 (1.7), 22 404 patients (47.3%) had an ATRIA (Anticoagulation and Risk Factors in Atrial Fibrillation) bleeding risk score of 5 or more, and 8890 patients (18.8%) had a history of hospitalization for bleeding. There were 2328 of 23 546 patients (9.9%) in the intervention group with initiation of OAC at 1 year compared with 2330 of 23 787 patients (9.8%) in the control group (adjusted OR, 1.01 [95% CI, 0.95-1.07]; *P* = .79).

**Conclusions and Relevance:**

Among a large population with AF with a guideline indication for OACs for stroke prevention who were randomized to a mailed educational intervention or to usual care, there was no clinically meaningful, numerical, or statistically significant difference in rates of OAC initiation. More-intensive interventions are needed to try and address the public health issue of underuse of anticoagulation for stroke prevention among patients with AF.

**Trial Registration:**

ClinicalTrials.gov Identifier: NCT03259373

## Key Points

**Question:**

Does a single educational mailing to a patient with atrial fibrillation and their clinician increase the use of oral anticoagulants for stroke prevention?

**Findings:**

This randomized clinical trial found no statistically significant increase in initiation of oral anticoagulants among patients with atrial fibrillation after a single educational mailing to patients and their clinicians.

**Meaning:**

More-intensive interventions are needed to address the public health issue of underuse of anticoagulation for stroke prevention among patients with atrial fibrillation.

## Teaser

This randomized clinical trial investigates whether an educational intervention directed to patients and their clinicians stimulates the use of oral anticoagulants in patients with atrial fibrillation (AF) who are not receiving oral anticoagulants (OACs).

## Introduction

Atrial fibrillation (AF) is the most common sustained arrhythmia.^1,2^ Patients with AF have a 4% to 5% annual incidence of stroke and are at a 5-fold to 7-fold higher risk of stroke than patients without AF.^3,4,5^ Strokes related to AF have higher morbidity and mortality than non-AF–related strokes,^6,7^ and AF-related strokes make up 15% to 20% of all strokes in the United States.^8^ Oral anticoagulants (OACs) decrease stroke risk by approximately 70%.^9,10^ There is a recommendation in the AF guidelines to use OACs for patients with a CHA_2_DS_2_-VASc (cardiac failure or dysfunction, hypertension, age 65-74 [1 point] or ≥75 years [2 points], diabetes, and stroke, transient ischemic attack or thromboembolism [2 points]–vascular disease, and sex category [female]) score of 2 or higher.^11,12^

Despite the existence of safe and effective pharmacologic treatments for stroke prevention among patients with AF, only 40% to 60% of patients with AF and a guideline indication for an OAC are treated with an OAC.^13,14,15,16^ Two-thirds of patients with AF who experience a stroke were not taking an OAC at the time of their stroke.^17^ The availability of safer and easier to use non–vitamin K OACs has only modestly increased overall OAC use among patients with AF.^18^ Patients emphasize the importance of stroke prevention and willingness to tolerate an increased risk of bleeding, while clinicians focus on bleeding risk and favor a higher stroke risk to warrant use of an OAC.^19^ Interviews with patients with AF indicate that clinicians often do not discuss the risk of stroke vs bleeding with use of an OAC.^20^

A method in current practice to increase the use of OACs is mailing educational materials directly to patients, with the goal of having them act as change agents. This method has been shown to increase the use of β-blockers after acute myocardial infarction but has not been evaluated for AF.^21^ The Implementation of a Randomized Controlled Trial to Improve Treatment With Oral Anticoagulants in Patients With Atrial Fibrillation (IMPACT-AFib) trial was a pragmatic randomized clinical trial embedded in health plans that participate in the US Food and Drug Administration’s (FDA) Sentinel System.

## Methods

### Study Oversight

The study was sponsored by the FDA through FDA-Catalyst. The trial was designed and led by a steering committee consisting of a patient representative and representatives from the participating health plans (Aetna, HealthCore/Anthem, Harvard Pilgrim Health Care, Humana, and Optum), the FDA, the Clinical Trials Transformation Initiative, the central study coordinating center (Harvard Pilgrim Health Care Institute), and a statistical coordinating center (Duke Clinical Research Institute). The Biomedical Research Alliance of New York institutional review board served as the single institutional review board and approved this study. A waiver of patient consent was obtained because contacting patients for consent would be an intervention by itself and might have affected the results of the trial, while the intervention was consistent with quality improvement initiatives already being performed by health plans, carried a low risk of harm, and placed no restrictions on the care of the control group. The Consolidated Standards of Reporting Trials (CONSORT) reporting guideline for randomized clinical trials was followed.

### Trial Design

IMPACT-AFib (ClinicalTrials.gov identifier: NCT03259373) was a prospective, multicenter, open-label, educational intervention randomized clinical trial conducted from September 25, 2017, to May 1, 2019 (trial protocol and statistical analysis plan in Supplement 1).^22^ Patients were randomized to receive an intervention of patient and clinician education (eFigure 1 and eFigure 2 in Supplement 2) either at the inception of the study (intervention) or after 1 year (the usual-care control). Patients and their treating clinicians were identified through health insurance claims data from commercially insured and Medicare Advantage populations.

### Study Population

The trial was conducted within FDA-Catalyst, which uses the FDA’s Sentinel System infrastructure for research. The FDA’s Sentinel Initiative was developed to assess the safety of approved medical products by using a common data model. Inclusion criteria were 2 or more diagnoses of AF (*International Classification of Diseases, Ninth Revision* [*ICD-9*] and/or *International Statistical Classification of Diseases and Related Health Problems, Tenth Revision* [*ICD-10*] codes) at least 1 day apart and with at least 1 diagnosis within 12 months of randomization; CHA_2_DS_2_-VASc score of 2 or greater; medical and pharmacy insurance coverage at randomization and for at least 12 months prior; and aged 30 years or older. Exclusion criteria included OAC dispensing (defined as 1 claim for an OAC dispensing by a pharmacy or 4 international normalized ratio test results) during the 12 months prior to randomization; conditions other than AF that required anticoagulation (mechanical prosthetic valve, history of deep venous thrombosis or pulmonary embolism); pregnancy in the past 6 months; any history of intracranial hemorrhage; hospitalization for any bleeding within 6 months; or P2Y12 antagonist use within 90 days. Patients were excluded if they disenrolled from the health plan between randomization and mailing or were not able to be included in research.

### Randomization

**Figure 1.**
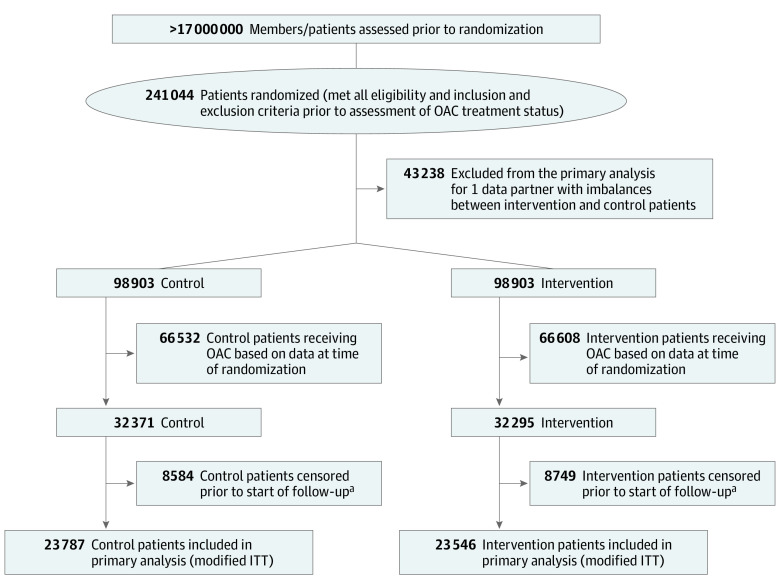
Flow Diagram of Primary Analysis Population ^a^Reasons for censoring: evidence of oral anticoagulant (OAC) treatment prior to start date based on more complete data, disenrolled earlier than originally captured in data (owing to changing in data over time), not able to be included in research owing to type of health plan, or request not to be contacted for research.

Patients were randomized 1:1 to intervention vs control. Concerns were raised about the ethics of identifying patients with AF who were not treated with an anticoagulant and then not notifying the patients or their clinicians of this information. On the advice of an ethics review panel, the decision was made to randomize patients with AF without the knowledge of their anticoagulation status. Anticoagulation status was evaluated in the intervention group only at the beginning of the study, while the anticoagulation status of the control group was identified retrospectively at the conclusion of the trial (Figure 1). Patients in the intervention cohort received a 1-time patient and clinician educational mailing at trial start (eFigure 1 and eFigure 2 in Supplement 2). The control cohort received usual care during the 12-month study period, and clinicians of these patients were mailed intervention material after the study period.

Among more than 17 million members enrolled in the 5 health plans, 241 044 were randomized after meeting all inclusion and exclusion criteria, including patients treated and not treated with OACs, from whom we identified the primary analysis subset of those not receiving OACs (determined at baseline for the intervention cohort and determined [retrospectively to match the baseline status] after 12 months in the control group).^22^ Missing baseline data necessary to contact patients or clinicians at 1 data partner resulted in imbalances between the intervention and control patients from that data partner; all patients from that data partner were excluded from the primary analysis. The primary analysis included 4 data partners (47 333 patients); the fifth data partner (12 772 patients) was included in a sensitivity analysis (eTable 1 and eFigures 3-5 in Supplement 2).

### Outcomes

The primary end point was the proportion of patients with at least 1 OAC dispensed (defined as 1 outpatient claim for an OAC dispensing or 4 international normalized ratio test results) during 12 months of follow-up. Dispensings typically covered 30 to 90 days of therapy. Prespecified secondary end points were rates of hospitalized ischemic stroke, transient ischemic attack, systemic embolism, hemorrhagic stroke, and combinations thereof; rate of hospitalized bleeding; all-cause in-hospital mortality rates; proportion of days covered by OAC dispensings; proportion of patients receiving an OAC at 12 months of follow-up; and health care use. Clinical end points were identified via *ICD-10* diagnosis codes. We assessed these end points 2 ways: only those indicated as the principal discharge diagnosis and, separately, diagnosis codes in any position.

### Statistical Analysis

Primary end point power calculations assumed that 33% of control patients would initiate an OAC and 5% higher OAC initiation with the patient and clinician interventions during a 12-month follow-up period. With a 30% attrition rate and 2-sided type I error of .05, approximately 10 000 patients were needed for 99% power. Stroke or transient ischemic attack was a secondary clinical outcome, and approximately 47 000 patients would provide 80% power to detect a 0.89% absolute reduction, assuming a 1-year incidence of 14.2% among control patients. The control risk of 14.2% was estimated in a separate population of 1 of the data partners. Initial power calculations assumed enrollment of 80 000 patients, which was based on preliminary work in the Sentinel database; however, a portion of these 80 000 patients were ineligible to participate in the randomized clinical trial.

Randomized patients not receiving an OAC at the mailing were the modified intention-to-treat (mITT) population—the primary analysis population. Patient-level data were maintained by the health plans. Health plan–level analyses were conducted via distributed SAS programs (version 9.4; SAS Institute Inc) and generated aggregate results. Inverse-variance–weighted mean fixed-effect meta-analyses were performed on effect sizes from health plan–level analyses.^23^ For each health plan study *i*, we calculated its weight *W_i_* = (*1/V_Yi_*), where *V_Yi_* was the within-study variance for effect size *Y_i_* from study *i*. The weighted mean (*M* *=* [∑*W_i_Y_i_*/∑*W_i_*]) over all studies and its variance (*V* = [1/∑*W_i_*]) were calculated. The 95% CI for the weighted mean effect was calculated using *M* ± 1.96 × *V*^1/2^. The *z* score (*M*/*V*^1/2^) tested the null hypothesis that common true effect was 0 (for a difference) or 1 (for a ratio). *P* value was calculated for 2-tailed test using standard normal distribution *z* score and results were deemed statistically significant at *P* < .05.

For health plan–level analyses, generalized estimating equations logistic regression model adjusting for clustering (statistical dependence) of patients from the same clinician compared the primary end point after adjusting for baseline risk factors prespecified in the statistical analysis plan (Supplement 1). The generalized estimating equation method used compound symmetric working correlation matrix and empirical (sandwich) SE estimates. Odds ratios (ORs) and 95% CIs were calculated with the control group as the reference. Log OR was the effect size in the meta-analyses. The primary end point was assessed using subgroups by sex, age, and CHA_2_DS_2_-VASc score.

Secondary clinical outcomes were analyzed at the health plan level using cumulative incidence rates from Kaplan-Meier product limit method. Cox proportional hazards regression with robust SEs accounted for clustering of patients within the same clinician assessed the intervention effect. The covariates were the same as above. Hazard ratios (HRs) and 95% CIs were estimated with the control group as the reference. Cumulative incidence rates and log HR were used as effect sizes in the meta-analyses.

## Results

### Baseline Characteristics

Among 47 333 patients (24 909 men [52.6%]) included in the primary mITT analysis, patients had a mean (SD) age of 77.9 (9.7) years, with 11 369 (24.0%) being 85 years of age or older and 4995 (10.6%) being 90 years of age or older (Table). The mean (SD) CHA_2_DS_2_-VASc score was 4.5 (1.7), 22 404 patients (47.3%) had an ATRIA (Anticoagulation and Risk Factors in Atrial Fibrillation) bleeding risk score of 5 or more, and 8890 patients (18.8%) had a history of hospitalization for bleeding. Patients from all regions of the country were well represented. Mean (SD) follow-up in the intervention group was 423.9 (132.2) days and in the control group was 425.0 (135.9) days.

**Table.** Baseline Characteristics of Participants

Characteristic	Participants, No. (%)	
	Intervention (n = 23 546)	Control (n = 23 787)
Age, mean (SD), y	77.8 (9.7)	77.9 (9.7)
Age, y		
<55	471 (2.0)	436 (1.8)
55-59	569 (2.4)	562 (2.4)
60-64	934 (4.0)	979 (4.1)
65-69	2341 (9.9)	2360 (9.9)
70-74	4642 (19.7)	4762 (20.0)
75-79	4814 (20.5)	4946 (20.8)
80-84	4182 (17.8)	3966 (16.7)
85-89	3169 (13.5)	3205 (13.5)
≥90	2424 (10.3)	2571 (10.8)
Sex		
Female	11 262 (47.8)	11 162 (46.9)
Male	12 284 (52.2)	12 625 (53.1)
Region		
New England	645 (2.7)	731 (3.1)
Mid-Atlantic	1162 (4.9)	1298 (5.5)
South Atlantic	14 340 (60.9)	14 286 (60.1)
Midwest	4723 (20.1)	4885 (20.5)
Mountain	1484 (6.3)	1470 (6.2)
Pacific	906 (3.9)	859 (3.6)
Unknown or missing	286 (1.2)	258 (1.1)
Prior conditions		
Hypertension	22 338 (94.9)	22 583 (94.9)
Diabetes	9671 (41.1)	9625 (40.5)
Peripheral vascular disease	6001 (25.5)	5981 (25.1)
Prior cerebrovascular disease	4944 (21.0)	4863 (20.4)
Heart failure	9451 (40.1)	9452 (39.7)
Myocardial infarction	2827 (12.0)	2744 (11.5)
CABG	3409 (14.5)	3500 (14.7)
Coronary stent	1205 (5.1)	1188 (5.0)
Dialysis	664 (2.8)	616 (2.6)
CHA_2_DS_2_-VASc score, mean (SD)	4.53 (1.7)	4.50 (1.7)
ATRIA bleedign risk core ≥5	11 165 (47.4)	11 239 (47.3)
History of hospitalization for bleeding	4409 (18.7)	4481 (18.8)

Abbreviations: ATRIA, Anticoagulation and Risk Factors in Atrial Fibrillation; CABG, coronary artery bypass graft; CHA_2_DS_2_-VASc, cardiac failure or dysfunction, hypertension, age 65-74 (1 point) or ≥75 years (2 points), diabetes, and stroke, transient ischemic attack or thromboembolism (2 points)–vascular disease, and sex category (female).

### Primary Outcome

**Figure 2.**
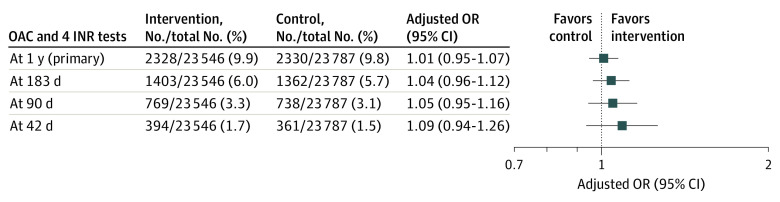
Primary Outcome Results Primary outcome of oral anticoagulant (OAC) initiation at 1 year, 183 days, 90 days, and 42 days with modified intention to treat for the primary analysis of 4 data partners. The forest plot for the odds ratios (ORs) is displayed on a log scale using a logarithmic axis. INR indicates international normalized ratio.

**Figure 3.**
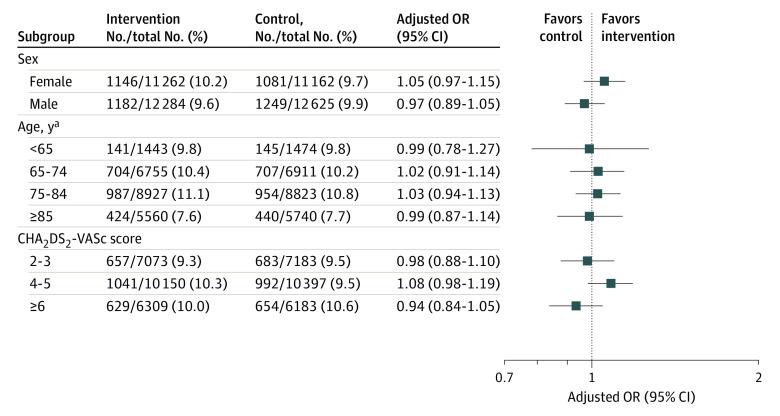
Subgroup Analysis Primary outcome of oral anticoagulant initiation at 1 year with modified intention to treat across prespecified subgroups for the primary analysis of 4 sites. The forest plot for the odds ratios (ORs) is displayed on a log scale using a logarithmic axis. CHA_2_DS_2_-VASc indicates cardiac failure or dysfunction, hypertension, age 65-74 (1 point) or ≥75 years (2 points), diabetes, and stroke, transient ischemic attack or thromboembolism (2 points)–vascular disease, and sex category (female). ^a^Meta-analysis data were used from 4 data partners.

At 1 year, there were 2328 of 23 546 patients (9.9%) in the intervention group and 2330 of 23 787 patients (9.8%) in the control group with initiation of an OAC (unadjusted OR, 1.01 [95% CI, 0.95-1.07]; *P* = .69; adjusted OR, 1.01 [95% CI, 0.95-1.07]; *P* = .79) (Figure 2). There was no statistically significant difference in OAC initiation at 42 days, 90 days, or 183 days. There was no statistically significant difference in OAC initiation at 1 year in any subgroup (Figure 3).

### Secondary Outcomes

Among patients initiating an OAC, the mean (SD) number of days taking an OAC was 166.5 (129.8) in the intervention group and 168.2 (130.1) in the control group (*P* = .85). Patients initiating an OAC were dispensed a sufficient dose of OAC for 36% of follow-up days, on average, and only 7.9% of patients initiated on OACs (420 of 5351) had at least 80% of follow-up time covered by an OAC—findings that were similar in both groups. There was no statistically significant difference in OAC persistence, with 54.7% of intervention patients (1135 of 2074) initiating an OAC and 56.5% of control patients (1183 of 2095) initiating an OAC remaining on an OAC at final follow-up (*P* = .30).

**Figure 4.**
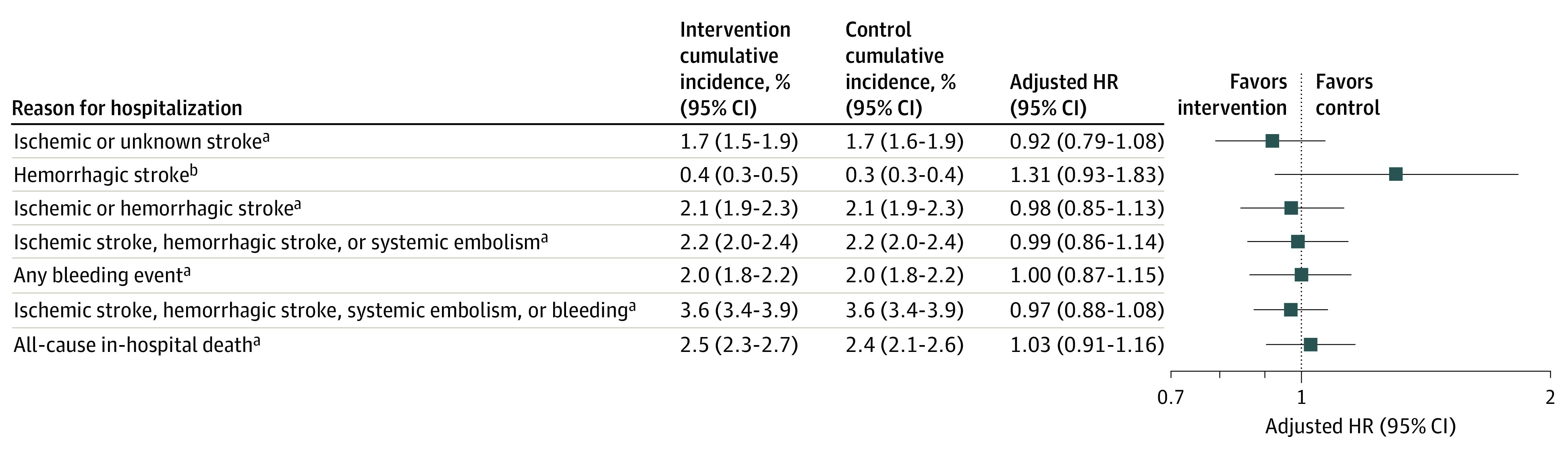
Secondary Clinical Outcomes Analysis Secondary clinical outcomes at 1 year with the modified intention-to-treat population from 4 sites. The forest plot for the hazard ratios (HRs) is displayed on a log scale using a logarithmic axis. ^a^Meta-analysis data were used from 4 data partners. ^b^Meta-analysis data were used from 3 data partners.

There were no statistically significant differences in ischemic stroke, hemorrhagic stroke, bleeding hospitalizations, or in-hospital all-cause mortality (Figure 4). The cumulative incidence of a principal diagnosis of ischemic stroke, hemorrhagic stroke, or systemic embolism was 2.2% (95% CI, 2.0%-2.4%) among the intervention group and 2.2% (95% CI, 2.0%-2.4%) among the control group (*P* = .60), while the cumulative incidence increased when using any claims position to 4.6% (95% CI, 4.3%-4.9%) among the intervention group and 4.9% (95% CI, 4.6%-5.2%) among the control group (*P* = .79).

Use of health care resources was similar in both groups (eTable 2 in Supplement 2). There were no statistically significant differences in the number of outpatient or emergency department encounters. There were statistically fewer, but a clinically similar number of, hospital admissions in the intervention (mean [SD] per patient, 0.43 [0.91]) vs control (mean [SD] per patient, 0.44 [0.96]) groups (*P* = .02).

### Sensitivity Analysis

There were 60 105 patients included in the mITT analysis among all 5 health plans. Baseline characteristics were similar to the primary mITT analysis (eTable 1 in Supplement 2). The sensitivity analysis also found no statistically significant difference in OAC initiation at 1 year (eFigure 3 in Supplement 2), including across subgroups (eFigure 4 in Supplement 2), or the secondary outcomes (eFigure 5 in Supplement 2).

## Discussion

The IMPACT-AFib trial found that there was no statistically significant or clinically meaningful difference in rates of OAC initiation at 1 year with a single educational mailing. The robustly negative conclusion points to 2 important actions. First, more intensive and/or targeted interventions to improve adherence to guideline recommendations are needed. Second, health plans that are currently using an unproven intervention, such as the mailings used in this trial, should consider alternative interventions to make better use of resources.

Changing practice, prescribing patterns, and patient adherence is difficult. The American Heart Association Get With The Guidelines–Atrial Fibrillation registry found that measurement and feedback was associated with higher rates over time of OAC prescription at hospital discharge.^24^ It has previously been shown that an intensive, hands-on, multifaceted intervention targeting clinicians in outpatient clinics, including measurement and feedback, resulted in a significant increase in the use of OAC in patients with AF, as well as a reduction in stroke.^25^ The goal of the IMPACT-AFib clinical trial was to study a less intensive and low-risk intervention that focused primarily on patients. However, the IMPACT-AFib trial found no difference in the rates of initiation of OAC within 1 year of a single patient and clinician educational mailing (adjusted OR, 1.01 [95% CI, 0.95-1.07]). This finding may be owing to use of a single intervention, when most studies showing success in guideline implementation have been multifaceted.^25,26^

A systematic review of interventions addressing the underuse of OAC for stroke prevention among patients with AF found that among 20 studies evaluated, interventions that targeted health care professional education and interventions with persuasive elements, including benchmarking to peer performance, were most successful.^27^ The systematic review also highlighted that nonrandomized studies were more likely to generate a positive result. In this regard, in the IMPACT-AFib trial, there was a 10% initiation of treatment among patients in the intervention group, and thus the negative result was evident only because of inclusion of an untreated control group, without which the intervention might have been declared successful. The use of a no-intervention control group occasioned debate about the ethics of identifying individuals who were not receiving evidence-based care, without notifying either the patients or their clinicians. The study did not impair care or in any way prevent initiation of OACs among control patients.^28^

Education, knowledge, and information in and of themselves do not necessarily translate to behavioral changes.^29^ One prior randomized trial in Europe found that an educational program did not improve adherence and persistence with OACs for stroke prevention in AF.^30^ Furthermore, written educational materials for patients tend to be less effective than education that is verbally shared by a clinician.^31^ Results are mixed, as a prior study with a simple direct-to-patient educational intervention that used 2 mailings 2 months apart was able to demonstrate a 4% absolute increase in adherence to β-blockers after myocardial infarction.^21^ Other forms of direct patient engagement, such as mobile health technology platforms, have been demonstrated in a cluster randomized clinical trial to be effective in reaching patients with AF, increasing OAC use, and improving outcomes.^32,33^ More than simple technological interventions are needed to facilitate behavioral change with clinicians, as electronic physician notifications have previously been shown to not affect prescribing patterns of OAC for patients with AF.^34^

The IMPACT-AFib trial advanced methods for pragmatic trials, demonstrating that it is feasible to use health plans’ existing infrastructure to identify eligible patients and clinicians, contact them, and then obtain outcomes. The Sentinel System’s distributed database, with a common data model, allowed multiple large national health plans to work together at a previously unavailable scale. This platform may be suitable to evaluate a wide array of interventions of the type that large health plans typically undertake, at a fraction of the cost of traditional randomized clinical trials. For the IMPACT-AFib trial, the cost was about $90 per patient for the 60 105 patients in the mITT analysis. However, evaluating more complex interventions and including informed consent would introduce challenges.

### Limitations

This study had some limitations. There were challenges and important lessons learned in use of the Sentinel Distributed Database platform in the IMPACT-AFib randomized trial.^35,36^ Certain patient characteristics, such as social determinants of health, were not available in the database. Although pharmacy claims data used for the primary end point of OAC initiation are highly accurate, there are limitations to the use of insurance claims to assess patient baseline characteristics and clinical outcomes. However, validation of *ICD-9* codes for stroke has shown a positive predictive value of 97% when limiting codes to the principal discharge diagnosis, while the positive predictive value was 84% with stroke in any claims position.^37^ The high positive predictive value of the codes when present does not address the number of events that might be undercoded or missed, but the reporting would be expected to be similar in both groups. Data from one of the data partners were excluded from the main analysis. However, the sensitivity analysis that did include data from that data partner did not show different findings than the main analysis.

The trial was unable to assess whether recipients received and read the educational information. Clinicians could have cared for patients included in both the intervention and control groups of the trial; however, the statistical methods accounted for this possibility by adjusting for clustering accounted for statistical dependence (meaning the clinician may have a tendency to treat all patients with an OAC regardless of their status in the intervention or control group). No adjustment was able to be made for multiple clinicians in the same practice, with some receiving intervention materials vs others only having patients in the control group. The AF guidelines in the US downgraded the recommendation for OAC use in women with a CHA_2_DS_2_-VASc score of 2 from class I to class IIa during the trial, but this occurred late in trial follow-up, and there was no change in OAC use in the early follow-up prior to the recommendation change.

## Conclusions

In a population with AF with a guideline-concordant indication for OAC for stroke prevention, there was no statistically significant or clinically meaningful difference in OAC initiation after a single educational mailing, compared with no mailing. Rates of OAC initiation were low in this high-risk population, with fewer than 1 in 10 patients initiating an OAC, underscoring the need to address undertreatment. More-intensive interventions are needed to address the public health issue of underuse of anticoagulation for stroke prevention in AF.

## Notes

Supplement 1.
Trial Protocol and Statistical Analysis Plan

Supplement 2. **eTable 1.** Baseline Characteristics of the Sensitivity Analysis Population **eTable 2.** Health Care Resource Utilization for the Modified Intention to Treat Population **eFigure 1.** Patient Educational Intervention Mailing Materials **eFigure 2.** Provider Educational Intervention Mailing Materials **eFigure 3.** Sensitivity Analysis of Primary Endpoint Results **eFigure 4.** Sensitivity Analysis of Primary Endpoint Subgroups **eFigure 5.** Sensitivity Analysis of Secondary Clinical Outcomes

Supplement 3.
Data Sharing Statement
