# Supplementary material for: Effect of Mailing Educational Material to Patients With Atrial Fibrillation and Their Clinicians on Use of Oral Anticoagulants: A Randomized Clinical Trial
Source: JAMA Netw Open. 2022 May 31;5(5):e2214321. doi: 10.1001/jamanetworkopen.2022.14321 (PMC9157265; doi:10.1001/jamanetworkopen.2022.14321)
Supplement: Supplement 1. — Trial Protocol and Statistical Analysis Plan [file jamanetwopen-e2214321-s001.pdf]

FDA-CATALYST PROTOCOL

IMPLEMENTATION OF A RANDOMIZED CONTROLLED TRIAL TO IMPROVE TREATMENT WITH ORAL ANTICOAGULANTS IN PATIENTS WITH ATRIAL FIBRILLATION (IMPACT-AFib)

Prepared by: Sean D. Pokorney, MD, MBA;[1] Noelle Cocoros, DSc, MPH;[2] Hussein Al-Khalidi, PhD;[3] Kevin Haynes, PharmD, MSCE;[4] Sana Al-Khatib, MD, MHS;[1] Crystal Garcia, MPH;[2] Jennifer Goldsack, MChem, MA, MBA, CPHQ;[5] Thomas Harkins, MPH, MA;[6] Robert Jin, MS;[2] Daniel Knecht, MD, MBA;[7] Daniel Lane, PharmD, PhD;[6] Nancy Lin, ScD;[8] Debbe McCall, BS, MBA;[9] Cheryl McMahill-Walraven, PhD, MSW;[7] Vinit Nair, BPharm, MS, RPh;[6] Emily O'Brien, PhD;[1] Lauren Parlett, PhD;[4] Jennifer Rymer, MD; [1] Ryan Saliga, BBA;[2] Yunping Zhou, MS;[6] Melissa Robb, RN;[10] Christopher B. Granger, MD;[1] Richard Platt, MD, MSc[2]

Author Affiliations: 1. Duke Clinical Research Institute and Division of Cardiology, Duke University Medical Center, Durham, NC; 2. Department of Population Medicine, Harvard Pilgrim Health Care Institute and Harvard Medical School, Boston, MA; 3. Duke Clinical Research Institute and Department of Biostatistics & Bioinformatics, Duke University Medical Center, Durham, NC; 4. HealthCore, Inc., Alexandria, VA; 5. Clinical Trials Transformation Initiative, Durham, NC; 6. Comprehensive Health Insights, Humana Inc., Louisville, KY; 7. Aetna: Aetna Informatics, Blue Bell, PA; 8. OptumInsight Inc, Waltham, MA; 9. Rowan Tree Perspectives Consulting Murrieta, CA; 10. Center for Drug Evaluation and Research, Office of Medical Policy, FDA, Silver Spring, MD

Version 1.0 March 1, 2017
Version 2.0 October 2, 2017
Version 3.0 November 24, 2017
Version 4.0 April 19, 2018
Version 5.0 March 7, 2019

The Sentinel System is sponsored by the U.S. Food and Drug Administration (FDA) to proactively monitor the safety of FDA-regulated medical products and complements other existing FDA safety surveillance capabilities. The Sentinel System is one piece of FDA's Sentinel Initiative, a long-term, multi-faceted effort to develop a national electronic system. Sentinel Collaborators include Data and Academic Partners that provide access to healthcare data and ongoing scientific, technical, methodological, and organizational expertise. The Sentinel Coordinating Center is funded by the FDA through the Department of Health and Human Services (HHS) Contract number HHSF223201400030I.

History of Modifications

Version	Date	Modification	By
V5.0	03/07/2019	• Updated text throughout to match decisions made during development of the statistical analysis plan (SAP)<br>• Annotated change in study design – early intervention mailing occurred in two waves (*VI. Study Design & Duration*)<br>• Updated Figures 1 and 2 for accuracy<br>• Added link to code list for inclusion and exclusion criteria<br>• Included reasons eligible members and providers might not be mailed the intervention (*IX. Method of Assigning Patients to an Intervention*)	IMPACT-AFib Workgroup
V4.0	04/19/2018	• Each Data Partner conducted its own mailing to trial participants. Due to the early intervention launch (start of follow-up) spanning multiple months:<br>o Clarified that the follow-up period for primary and secondary objectives will be calculated based on the date on which at least 80% of eligible study participants have at least 12 months of follow-up time<br>o Clarified that the follow-up period for exploratory objectives will be calculated based on the date on which at least 80% of eligible study participants have at least 24 months of follow-up time<br>o Noted that all possible person-time will be used to assess outcomes (*VI. Study Design & Duration*)<br>• Updated Figures 1 and 2 for accuracy	IMPACT-AFib Workgroup
V3.0	11/03/2017	• Updated anticipated trial size and power calculations<br>• Included more detail about provider identification process for mailing (*IX. Method of Assigning Patients to an Intervention)*<br>• Clarified population eligible for primary analyses (*XIV. Statistical Considerations, B. Populations for Analysis*)	IMPACT-AFib Workgroup

V2.0	10/02/2017	• Clarified descriptions of the early and delayed intervention arms, including timing of mailings; noted that both arms include usual care and reiterated that those with recent OAC treatment will be excluded from mailings<br>• Updated schematic diagrams based on improved descriptions of the early and delayed interventions<br>• Updated Sentinel-CTTI partnership objective (*V. Study Objectives, C. Exploratory Objectives*)<br>• Clarified medical and pharmacy coverage requirement (*VII. Study Population, A. Inclusion Criteria)*<br>• Included more details about sample size and power calculations (*XIV. Statistical Considerations, A. Sample Size and Power Calculations*)<br>• Added rationale for using INR tests/values as a proxy for anticoagulant dispensing(s) (*VII. Study Population*)<br>• Added description of patient written education materials and clarified that information regarding top misperceptions will be provided for providers (*VIII. Interventions*)<br>• Noted that feedback from the provider response mailers will not be linked to specific patients or providers (*XVI. Statistical Analyses, B. Responses from Providers*)<br>• Made many less substantive changes in the interests of completeness, accuracy, and flow	IMPACT-AFib Workgroup

FDA-Catalyst Protocol

Implementation of a Randomized Controlled Trial to Improve Treatment with Oral Anticoagulants in Patients with Atrial Fibrillation (IMPACT-AFib)

Table of Contents

I. PROTOCOL SYNOPSIS 1
II. INTRODUCTION 6
III. RESEARCH HYPOTHESIS 7
IV. STUDY RATIONALE 7
V. STUDY OBJECTIVES 7

A. PRIMARY OBJECTIVE 7
B. SECONDARY OBJECTIVE 8
C. EXPLORATORY OBJECTIVES 8

VI. STUDY DESIGN AND DURATION 8
VII. STUDY POPULATION 11

A. INCLUSION CRITERIA 11
B. EXCLUSION CRITERIA 12

VIII. INTERVENTIONS 12

A. PATIENT-LEVEL INTERVENTIONS (EARLY INTERVENTION ARM) 12
B. PROVIDER-LEVEL INTERVENTIONS (EARLY INTERVENTION AND DELAYED INTERVENTION ARM) 12

IX. METHOD OF ASSIGNING PATIENTS TO AN INTERVENTION 13
X. ETHICAL CONSIDERATIONS 14
XI. INSTITUTIONAL REVIEW BOARD/INDEPENDENT ETHICS COMMITTEE 14
XII. INFORMED CONSENT 14
XIII. INDEPENDENT ADVISORY COMMITTEE 15
XIV. STATISTICAL CONSIDERATIONS 15

A. SAMPLE SIZE AND POWER CALCULATIONS 15

1. Primary Endpoint 15
2. Important Secondary Analysis of Stroke or TIA 16

B. POPULATIONS FOR ANALYSIS 17

XV. OUTCOME DEFINITIONS 17
XVI. STATISTICAL ANALYSES 18

A. DEMOGRAPHICS AND BASELINE CHARACTERISTICS 18
B. RESPONSES FROM PROVIDERS 18
C. EFFECTIVENESS ANALYSES 18

XVII. DISSEMINATION PLAN OVERVIEW 19
XVIII. REFERENCES 20

I. PROTOCOL SYNOPSIS

**Protocol Title:**	**IM**plementation of a randomized controlled trial to im**P**rove treatment with oral **A**nti**C**oagulan**T**s in patients with **A**trial **Fib**rillation (IMPACT-AFib)
**Research Hypothesis:**	Education on stroke prevention in atrial fibrillation (AF) among AF patients and their providers can result in increased use of oral anticoagulants (OAC) for stroke prevention among those AF patients with guideline-based indications for oral anticoagulation (CHA_2DS_2-VASc score of 2 or greater)
**Study Schema and Length of Trial:**	Early patient and provider* education interventions versus usual care (with delayed provider* education intervention at the date on which at least 80% of eligible study participants have at least 12 months of follow-up time). Duration of follow-up for the primary outcome (OAC treatment) will be from the date that the early intervention materials are mailed through the date on which at least 80% of eligible study participants have at least 12 months of follow-up time; secondary outcomes will also be evaluated at this time.
**Study Objectives:**	**Primary:** evaluate the effect of the patient and provider education interventions (versus usual care with delayed provider education intervention) on the proportion of patients with evidence of at least one OAC prescription fill (defined as one OAC dispensing or 4 INR tests) over the course of the follow-up through the date on which at least 80% of eligible study participants have at least 12 months of follow-up time.<br><br>**Secondary:** evaluate the impact on outcomes of the patient and provider education interventions over the course of the follow-up through the date on which at least 80% of eligible study participants have at least 12 months of follow-up time:<br>1. Incident rate of stroke or transient ischemic attack (TIA) hospitalization<br>2. Incident rate of hospitalization for stroke<br>3. Time to first OAC prescription fill<br>4. Proportion of days covered by OAC prescription fills<br>5. Proportion of patients actively on OAC at 12 months of follow-up<br>6. Incident rate of hospitalization for any bleeding<br>7. All-cause in-hospital mortality rates

*Where it is possible to identify an individual provider. See Section IX. Method of Assigning Patients to an Intervention for further information.

	8. All-cause mortality rates among patients with accurate out-of-hospital mortality data (such as Medicare Advantage patients)<br>9. Health care utilization for AF patients, which would be reported as counts of number of health care utilization events (outpatient visits, days hospitalized, number of emergency department visits, etc.)<br><br>**Exploratory:**<br><br>10. Evaluate the effect of the education interventions on the primary and secondary endpoints over the course of the follow-up through the date on which at least 80% of eligible study participants have at least 24 months of follow-up time.<br>11. Explore the Clinical Trials Transformation Initiative (CTTI) and the FDA supported Sentinel Data Partners' ability to successfully conduct a pragmatic trial to answer important questions to improve public health
**Study Design:**	Prospective, randomized, open-label education intervention trial. Patients with AF and a CHA_2DS_2-VASc score of 2 or greater will be randomized in a 1:1 ratio to (a) the early intervention arm: early patient and provider educational interventions for those patients identified at the time of randomization and (b) the delayed intervention arm: usual care followed by provider education intervention 12 months after at least 80% of early intervention mailing has occurred (eligibility status of these patients will be assessed at time of delayed mailing). All inclusion criteria, exclusion criteria, and outcomes will be determined through claims data. The primary outcome is the proportion of AF patients with evidence of at least one OAC prescription fill over the course of the follow-up through the date on which at least 80% of eligible study participants have at least 12 months of follow-up time. A total of approximately 80,000 patients will be enrolled within multiple major health plans across the United States. Follow-up for the primary outcome and secondary outcomes will be assessed 12 months after the date on which at least 80% of eligible early intervention participants were mailed the early intervention materials. The randomization will be performed by the central coordinating center (Harvard Pilgrim), and statistical analyses will be performed by the Duke Clinical Research Institute. A second exploratory assessment of the primary and secondary endpoints will be performed once at least 80% of participants have at least 24 months of follow-up time to assess the durability and longer-term outcomes of the effect of the education intervention. Because the Sentinel Distributed Database will be used for follow-up information, and this information is refreshed approximately quarterly and this is done on separate timetables for the different health plans, it is likely that when at least the required follow-up

	time is available for at least 80% of people, there will be more than 12 or 24 months of follow-up for over 80% of people. All possible person-time will be used to assess participants' outcomes (patients will have different duration of follow-up).
**Accrual Goal (Total number of participants):**	Approximately 80,000 patients across all participating Data Partners (Aetna, Harvard Pilgrim, HealthCore, Humana, and Optum)
**Accrual Rate (Number of participants expected per month):**	All patients and providers will be randomized prior to the initiation of the early intervention. The enrollment date for all patients (early and delayed intervention patients) will be the date that the letters are mailed out to early intervention patients and providers by their respective health plans.
**Inclusion Criteria:**	1. Two or more diagnoses of AF (ICD-9 and/or ICD-10 codes) at least one day apart and with at least one diagnosis within the last 12 months prior to the last date in the current approved data used for cohort identification<br>2. CHA_2DS_2-VASc score of 2 or greater<br>3. Medical and pharmacy insurance coverage of at least the prior year as identified via administrative claims databases of one of the participating Data Partners as of the date of randomization<br>4. Age 30 years or greater as of the last date in the current approved data used for cohort identification
**Exclusion Criteria:**	1. Evidence of OAC medication fill during the 12 months prior to randomization (determined at randomization for the early intervention cohort and 12 months post-randomization for the delayed intervention cohort)<br>2. Conditions other than AF that require anticoagulation, including treatment of deep venous thrombosis, pulmonary embolism, or ever having had a mechanical prosthetic heart valve prior to the last date in the current approved data used for cohort identification<br>3. Pregnancy within 6 months of the last date in the current approved data used for cohort identification<br>4. Any known history of intracranial hemorrhage prior to the last date in the current approved data used for cohort identification<br>5. Hospitalization for any bleeding within the last 6 months of the last date in the current approved data used for cohort identification<br>6. Patients with recent P2Y12 antagonist use (i.e. clopidogrel, prasugrel, ticlopidine, or ticagrelor) within 90 days of the last date in the current approved data used for cohort identification
**Criteria for Evaluation (Effectiveness, safety, stopping rules, etc.):**	The primary outcome is evidence of at least one OAC prescription fill through the date on which at least 80% of eligible study participants have at least 12 months of follow-up time.

**Patient-Level Interventions (early intervention arm):**	• Letters to patients that (1) explain to the patient that he or she appears to have AF, characterize the risk of stroke, and emphasize that although there may be a medical reason, the patient does not seem to be on an anticoagulant and (2) encourage the patient to discuss this with his or her provider to ask if he or she might benefit from OAC therapy to prevent stroke<br>• Written education materials<br>• Website with the patient-focused information contained in the letters to patients
**Provider-Level Interventions (early intervention and delayed intervention arms):**	• Letters to providers:<br>○ Early intervention letters to providers that explain this project, the nature of the problem, and identify a list of the provider's patients who have been contacted, as the provider and patient letters will be sent at approximately the same time; describe evidence and guidelines regarding oral anticoagulation<br>○ Delayed intervention letters to providers that explain this project, the nature of the problem, and identify a list of the provider's patients who are at risk for stroke and have not been treated with an oral anticoagulant; describe evidence and guidelines regarding oral anticoagulation<br>• Response mailer that gives the provider the opportunity to share the rationale for his or her patient(s) not being on OAC<br>• Web portal with access to clinical practice guidelines, decision support tools including from professional societies, podcasts, and case studies targeted at improving the appropriate use of OAC for AF<br>• Information sheet describing top misperceptions of barriers to OAC use<br>○ Misperception of benefit and risk of aspirin: it is neither safe nor effective<br>○ Misperception around risk of resuming OAC (months) after bleeding<br>○ Misperception regarding risk of OAC regarding patients who fall<br>○ Opportunities with novel OACs (NOACs) for patients who have not tolerated warfarin<br>○ Concern about lack of an antidote for the NOACs
**Statistics and Power Calculations:**	Primary outcome: proportion of AF patients with evidence of at least one OAC prescription fill through the date on which at least 80% of eligible study participants have at least 12 months of follow-up time<br><br>Sample size and power determination assumptions:

	• 33% OAC initiation rate in the delayed intervention arm over the first year of the study<br>• 38% OAC initiation rate in the early intervention arm (a 5% improvement in OAC initiation over the 33% OAC initiation expected in the delayed intervention arm over 1-year follow-up)<br>• 1-year attrition rate: 30% dropout or lost-to-follow-up<br>• Two-sided type I error of 0.05<br>• Roughly 10,000 patients will yield more than 99% power to detect a 5% absolute difference<br><br>Secondary outcome: stroke or transient ischemic attack (TIA) over the course of the follow-up through the date on which at least 80% of eligible study participants have at least 12 months of follow-up time. A study with approximately 80,000 patients is reasonably powered to detect a reduction in stroke or TIA under assumptions listed below:<br>• 1-year stroke or TIA rate: 18% among patients not treated with OAC<br>• 1-year stroke or TIA rate: 7% among patients treated with at least 1 OAC fill<br>• Duration of follow-up: 1 year<br>• 33% of delayed intervention patients will have at least 1 fill of OAC, meaning the 1 year stroke or TIA rate in the delayed intervention group would be 14.4%<br>• If 38% of early intervention patients have at least 1 fill of OAC (meaning the 1 year stroke or TIA rate in the early intervention group would be 13.82%, i.e., an absolute reduction of 0.55%), the study will have 46% power to detect this 0.55% reduction. However, if 40.5% of early intervention patients have at least 1 fill of OAC (meaning the 1 year stroke or TIA rate in the early intervention group would be 13.54%, i.e., an absolute reduction of 0.83%), the study will have 80% power to detect this 0.83% reduction.<br>• 1-year attrition rate: 30% dropout or lost-to-follow-up<br>• Two-sided type I error of 0.05<br>• The sample size has 80% power to detect a 0.5% absolute reduction in stroke, assuming a cumulative 1-year incidence of stroke of 4.2% in control (delayed intervention arm) patients and 3.7% in intervention (early intervention arm) patients. The assumption is that patients not on oral anticoagulation have an annual stroke rate of 5%, and stroke will be reduced by 50% (HR=0.5) in the treated (anticoagulated) population. The 80% power requires that 52% of early intervention arm patients are treated at 1-year as compared to 33% in the control arm.

II. INTRODUCTION

Atrial fibrillation (AF) is the most common arrhythmia seen in clinical practice, accounting for one-third of rhythm-related hospitalizations.[1] The prevalence of AF in the United States is 1% overall and more than 10% in people over age 80 years, with approximately 70% of cases in patients between 65 and 85 years of age.[2] As Americans age, the number of patients with AF is expected to increase 150% by 2050, with more than 50% of patients being octogenarians or older.[3-8] The increasing burden of AF is expected to lead to a higher incidence of stroke, as patients with AF have a five to seven fold greater risk of stroke than the general population.[9-11] The incidence of stroke in AF patients is 4 to 5% per year.[12] Patients with a stroke due to AF have worse prognoses than patients with stroke without AF.[13,14] Oral anticoagulants (OACs) are an important therapy for patients with AF as they decrease the rate of stroke by more than two-thirds.[15]

The clinical benefit of warfarin is only seen in patients who consistently maintain their international normalized ratio (INR) values within the therapeutic range (2.0-3.0).[16] The median times in the therapeutic range in the ARISTOTLE[17], ROCKET AF[18], RE-LY[19], and ENGAGE[20] trials were 62%, 55%, 64%, and 68%, respectively. As part of quality improvement efforts, there is significant interest in measuring and improving performance of physicians in prescribing anticoagulation for patients with AF. The $CHADS_2$ and CHA_2DS_2-VASC scores have been developed to support providers in identifying patients with AF who are at an increased risk for stroke.[21,22] Paradoxically, anticoagulation use is no higher, or declines, as the risk of stroke, as measured by these scores, increases.[23] This is in large part due to the fact that physicians and patients are concerned about the risk of bleeding in higher-risk populations, and they may underappreciate the net benefit from anticoagulants, including stroke prevention. In any case, approximately 40-60% of patients with AF at risk of stroke are not prescribed anticoagulation therapy.[15,24-30] This translates to over 50,000 preventable strokes each year in the United States with a number needed to treat of approximately 25 patients to prevent 1 stroke.

The specific reasons for not using an anticoagulant in eligible AF patients are poorly defined. The AVERROES trial provided some insight into reasons for patients deemed "unsuitable for warfarin" with 42% unable to maintain therapeutic INR, 43% unlikely to monitor INR, and 37% refusing warfarin.[31] The reasons for underuse of oral anticoagulation are likely related to three types of barriers: patient-level barriers (i.e. overestimation of risk of bleeding, underestimation of risk of stroke, misperceptions of adverse events from OACs), provider-level barriers (i.e. overestimation of risk of bleeding, underestimation of risk of stroke, misperceptions of adverse events from OACs, misperceptions about a patient's fall risk, misperceptions of the benefits of aspirin in stroke prevention), and system-level barriers (i.e. lack of time in a clinic visit to discuss stroke prevention, access to care such as an anticoagulation clinic, cost of OACs, feasibility of warfarin monitoring).

Another issue driving under-treatment with oral anticoagulants is poor medication adherence, which may be driven by patient barriers or system-level barriers. Medication non-adherence is defined as a patient's passive failure to follow a prescribed drug regimen. An average of 50% of patients are non-adherent to their prescribed treatment regimens for chronic diseases, such as AF.[32] Non-adherence rates are similar across disease states, treatment regimens, and age groups, with the first several months of therapy characterized by the highest rates of discontinuation.[33] Interventions to support patient management of medications have fallen short, and sustainable adherence rates have not improved.[34,35]

The cost to the health care system of hospitalizations due to medication non-adherence for all disease states may be as high as approximately $100 billion annually.[36] Patient-provider communication regarding medication use is poor, and opportunities to document and provide feedback on metrics for medication adherence are not readily available in EMR applications.[37] Meaningful and measurable improvements in adherence require a comprehensive, multifaceted intervention in which all participants, including physicians, play an active part.

III. RESEARCH HYPOTHESIS

Among AF patients with guideline indications for OAC for stroke prevention who have not received any OAC dispensing for at least one year, an education intervention at the patient-level and the provider-level will increase the proportion of patients started on OAC for stroke prevention.

IV. STUDY RATIONALE

Preliminary data from three Sentinel Data Partners between 1/1/2006 and 6/30/2015, identified 231,696 patients (1.4%) with at least 2 diagnoses of AF (ICD-9 codes of 427.31 or 427.32) over the study period, among 16.2 million covered lives. Within the 231,696 AF patients, there were 201,882 with a CHA_2DS_2-VASc score of 2 or greater (based on administrative claims data in the Sentinel Distributed Database), which is an American Heart Association (AHA)/American College of Cardiology (ACC) guideline indication for OAC. Over the study period from 2006 through 2015, 52% (n=105,256) patients filled at least one prescription for anticoagulation, meaning that 48% of patients with CHA_2DS_2-VASc score of 2 or greater never had a pharmacy claim submitted for an OAC during the study period. While there may be clinical or health system related reasons for not treating some of these patients, this apparent significant gap of guideline-based adherence is a public health issue of interest. The presented protocol is an opportunity to test the potential for developing and testing a health plan (Data Partner) based education intervention to improve the quality of care.

V. STUDY OBJECTIVES

A. PRIMARY OBJECTIVE

The primary objective is to evaluate the effect of the patient and provider education interventions (versus usual care with delayed provider education intervention) on the proportion of patients with evidence of at least one OAC prescription fill (defined as one OAC dispensing or 4 INR tests) over the course of the follow-up through the date on which at least 80% of eligible study participants have at least 12 months of follow-up time.

B. SECONDARY OBJECTIVE

The secondary objectives are to evaluate the impact on outcomes of the patient and provider education interventions over the course of the follow-up through the date on which at least 80% of eligible study participants have at least 12 months of follow-up time:

1. Incident rate of stroke or transient ischemic attack (TIA) hospitalization
2. Incident rate of hospitalization for stroke
3. Time to first OAC prescription fill
4. Proportion of days covered by OAC prescription fills
5. Proportion of patients actively on OAC at 12 months of follow-up
6. Incident rate of hospitalization for any bleeding
7. All-cause in-hospital mortality rates
8. All-cause mortality rates among patients with accurate out-of-hospital mortality data (such as Medicare Advantage patients)
9. Health care utilization for AF patients, which would be reported as counts of number of health care utilization events (outpatient visits, days hospitalized, number of emergency department visits, etc.)

C. EXPLORATORY OBJECTIVES

The exploratory objectives are to:

1. Evaluate the effect of the early and delayed education interventions on primary and secondary endpoints over the course of the follow-up through the date on which at least 80% of eligible study participants have at least 24 months of follow-up time.
2. Explore the CTTI and the FDA supported Sentinel Data Partnership's ability to successfully conduct a pragmatic trial to answer important questions to improve public health.

VI. STUDY DESIGN AND DURATION

The study is a prospective, randomized, and open-label education intervention trial. Patients with AF and a CHA_2DS_2-VASc score of 2 or greater will be randomized in a 1:1 ratio to an early intervention cohort and a delayed intervention cohort within each participating health plan. The definition for OAC medication fill will be an OAC medication dispensing or at least 4 INR tests in the claims data.[1] The claims records of the patients randomized to the early intervention cohort will then be linked to "fresh" (i.e. about 1-4 months old) pharmacy claims data at the time of randomization. Patients without evidence of an OAC medication fill during the 12 months prior to randomization will be included in the patient-level and provider-level early educational intervention (patients randomized to this early intervention with evidence of an OAC medication fill during the 12 months prior to randomization will be excluded from the trial.). In addition to usual care, these patients and their providers[2] will receive a

---

[1]Not all OAC dispensings are well captured in pharmacy claims, particularly, for warfarin. INR tests are assumed indicative of OAC fills that were not billed through the claims. 4 INR tests or values within a 12-month period will be used as a proxy since that is roughly the number of tests administered in the process of stabilizing dose.
[2]See Section IX. Method of Assigning Patients to an Intervention for detail regarding provider identification. In certain circumstances, a member or provider may not receive the one-time mailing.

one-time mailing at trial start. There will be two waves of mailings for the early intervention cohort at most sites due to the practical challenges of claims data: the patients are assigned to wave 1 if they have a provider easily identified in the data (i.e., the provider associated with the most recent AF diagnosis is an individual provider), and they are assigned to wave 2 if it is difficult to identify a provider (e.g., the first identified provider is a facility). Follow-up time will start on the date of the respective wave 1 and wave 2 mailings for the early intervention patients

The delayed intervention cohort will receive usual care over the initial study period. After the date on which at least 80% of eligible study participants have at least 12 months of follow-up time, "fresh" pharmacy claims data for the delayed intervention cohort that was generated and locked at the time of randomization will be used to assess trial eligibility, and those patients without evidence of an OAC medication fill during the 12 months prior to randomization will be included in the primary and secondary analyses as the delayed intervention arm. Patients randomized to the delayed intervention arm with evidence of an OAC medication fill during the 12 months prior to randomization will be excluded from the trial and will not be included in analyses. The baseline characteristics of the delayed intervention patients will be examined at the same time point as the early intervention patients, meaning at the time of randomization. The primary outcome is a comparison of the proportion of patients not on OAC during the 12 months prior to randomization, who were started on OAC over the course of the follow-up through the date on which at least 80% of eligible study participants have at least 12 months of follow-up time in the early versus the delayed intervention arm. A total of approximately 80,000 patients (randomized 1:1) across all participating Data Partners (Aetna, Harvard Pilgrim, HealthCore, Humana, and Optum) will be enrolled from participating Data Partners across the United States. Similar to the early intervention cohort, the patients in the delayed intervention cohort will be assigned to wave 1 or wave 2 ("pseudo" wave assignments) depending on the difficulty in identifying the patient's provider (the intent is to handle them the same way as was done for the early intervention arm). The follow-up for the delayed intervention patients will start on the date the wave 1 or 2 mailings take place for a given Data Partner's early intervention cohort. The follow-up time for the primary outcome will be 12 months from the date at which at least 80% of eligible study participates are enrolled (date on which early intervention materials are mailed).

The providers of patients in the delayed cohort who did not receive OAC medication during the course of the 12-month study period and meet our inclusion criteria will receive the delayed intervention: the provider-only education intervention, a one-time mailing administered 12 months after at least 80% of early intervention mailing have occurred (patients will not receive any educational materials unless no provider can be identified for a mailing). We intend to assess the primary and secondary endpoints again once at least 80% of patients have at least 24 months of follow-up time to assess the durability and longer-term outcomes of the effect of the patient- and provider-level education intervention, as well as the use of OAC following the delayed provider-level education intervention. However, as this second assessment is exploratory, we may not conduct these analyses if the results of the primary outcome are null.

Because the Sentinel Distributed Database will be used for follow-up information, and this information is refreshed approximately quarterly and this is done on separate timetables for the different health plans, it is likely that when at least the required follow-up time is available for at least 80% of people, there will be more than 12 or 24 months of follow-up for over 80% of people. All participants' outcomes will be assessed using all possible person-time; patients will have different duration of follow-up and that will be accounted for in the analyses.

A schematic diagram below shows the design of the early intervention period of the study over the course of the follow-up through the date on which at least 80% of eligible study participants have at least 12 months of follow-up time:

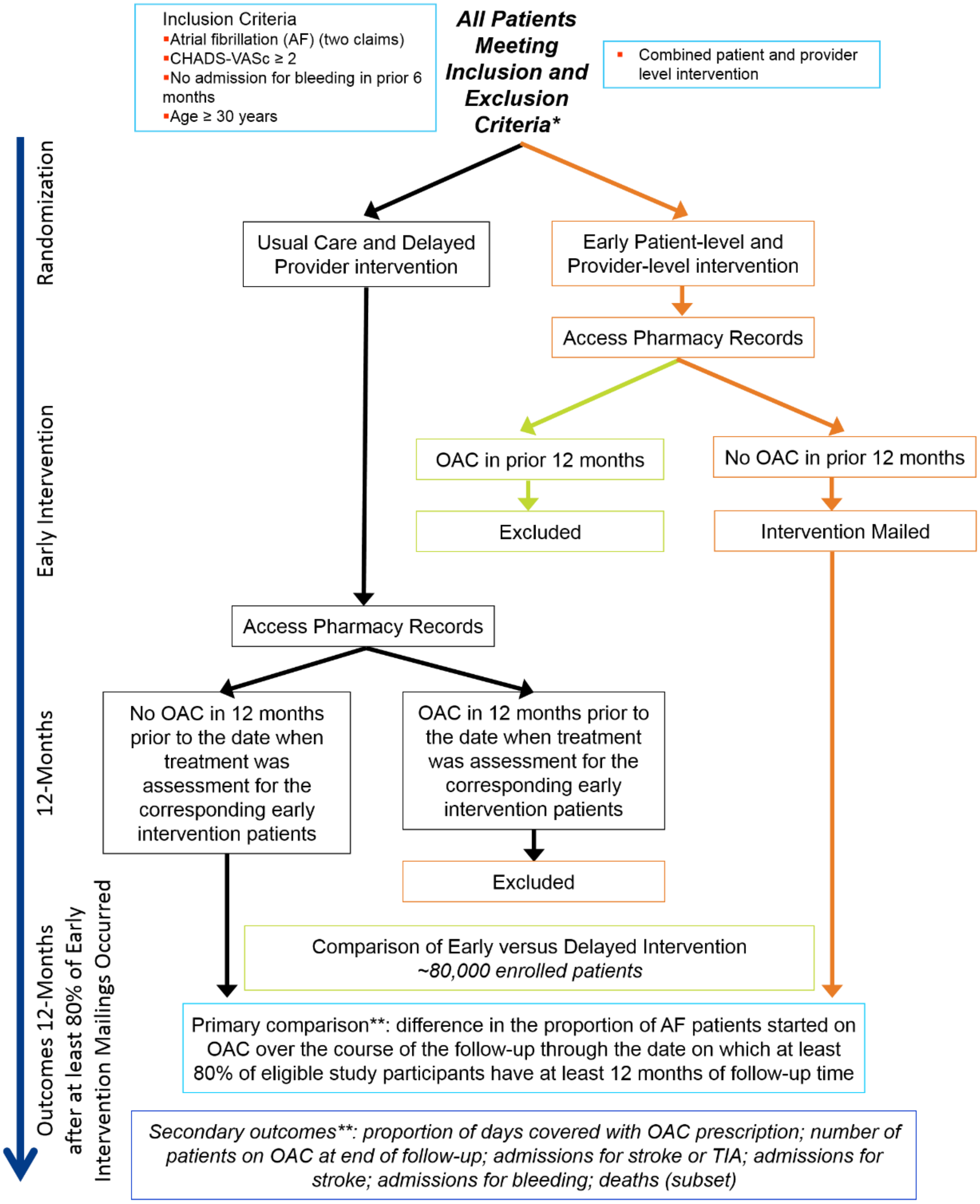

*Baseline characteristics of delayed and early intervention cohorts will be taken from the same time point at randomization from a dataset that is archived at randomization, while exclusion criteria for evidence of OAC medication fill or P2Y12 antagonist use was determined at randomization for the early intervention cohort and approximately 12 months post-randomization for the delayed intervention cohort.
**All possible person-time will be used to assess participants' outcomes (patients will have different duration of follow-up).

For analysis, treatment status is at time of randomization or corresponding early intervention mailing; for the delayed intervention mailing it is prior to mailing.

A schematic diagram below shows the design of the delayed intervention portion of the study:

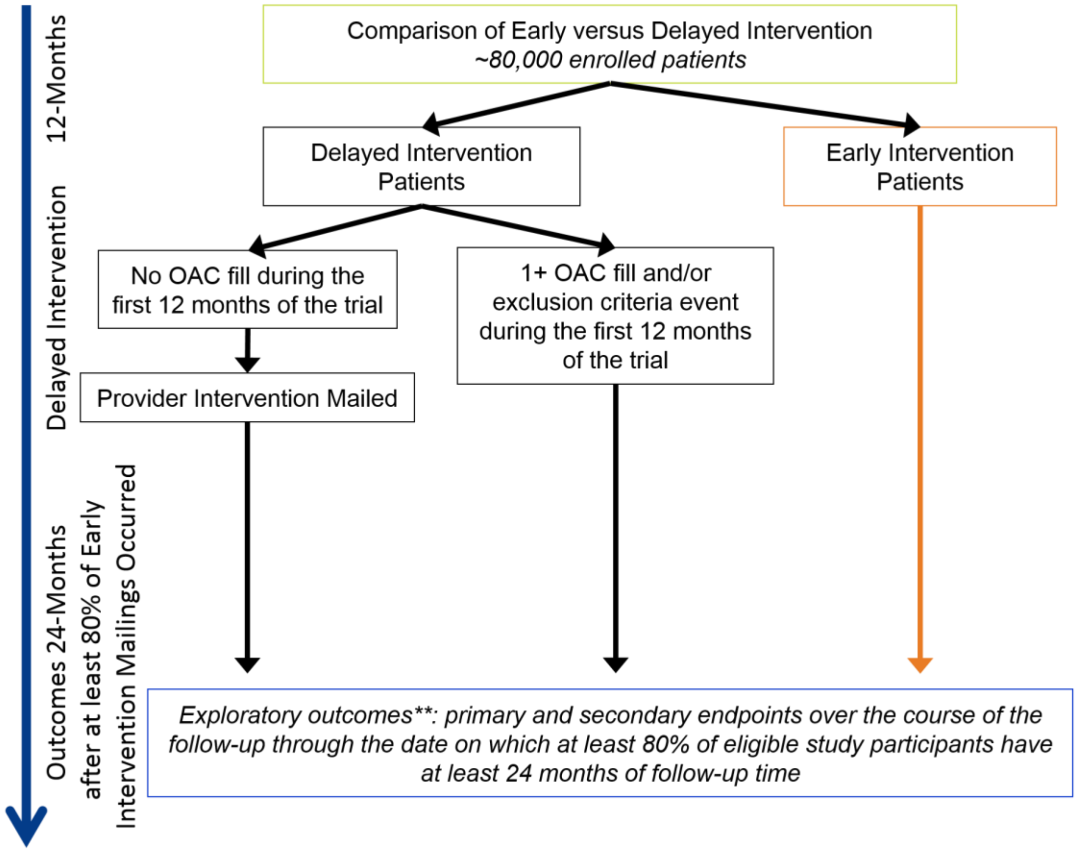

*All possible person-time will be used to assess participants' outcomes (patients will have different duration of follow-up).

For analysis, treatment status is at time of randomization or corresponding early intervention mailing; for the delayed intervention mailing it is prior to mailing.

VII. STUDY POPULATION

All inclusion and exclusion criteria will be determined by claims data. For entry into the study, the following criteria will be met:

A. INCLUSION CRITERIA

1. Two or more diagnoses of AF (ICD-9 and/or ICD-10 codes) at least one day apart and with at least one diagnosis within the last 12 months prior to the last date in the current approved data used for cohort identification
2. CHA_2DS_2-VASc score of 2 or greater
3. Medical and pharmacy insurance coverage of at least the prior year as identified via administrative claims databases of one of the participating Data Partners as of the date of randomization
4. Age 30 years or greater as of the last date in the current approved data used for cohort identification

B. EXCLUSION CRITERIA

1. Evidence of OAC medication fill (defined as 1 OAC dispensing or 4 INR tests) during the 12 months prior to randomization (determined at randomization for the early intervention cohort and 12 months post-randomization for the delayed intervention cohort)
2. Conditions other than AF that require anticoagulation, including treatment of deep venous thrombosis, pulmonary embolism, or ever having had a mechanical prosthetic heart valve prior to the last date in the current approved data used for cohort identification
3. Pregnancy within 6 months of the last date in the current approved data used for cohort identification
4. Any known history of intracranial hemorrhage prior to the last date in the current approved data used for cohort identification
5. Hospitalization for any bleeding within the last 6 months of the last date in the current approved data used for cohort identification
6. Patients with recent P2Y12 antagonist use (i.e. clopidogrel, prasugrel, ticlopidine, or ticagrelor within 90 days of the last date in the current approved data used for cohort identification

The complete code list for inclusion and exclusion criteria is accessible on the Sentinel website (https://www.sentinelinitiative.org/FDA-catalyst/projects/implementation-randomized-controlled-trial-improve-treatment-oral-anticoagulants-patients).

VIII. INTERVENTIONS

A. PATIENT-LEVEL INTERVENTIONS (EARLY INTERVENTION ARM)

- Letters to patients that (1) explain to the patient that he or she appears to have AF, characterize the risk of stroke, and emphasize that although there may be a medical reason, the patient does not seem to be on an anticoagulant and (2) encourage the patient to discuss this with his or her provider to ask if he or she might benefit from OAC therapy to prevent stroke.
- Written education materials about AF and anticoagulants, including answers to commonly asked questions
- Website with the patient-focused information contained in the letters to patients

B. PROVIDER-LEVEL INTERVENTIONS (EARLY INTERVENTION AND DELAYED INTERVENTION ARM)

- Letters to providers:
    - Early intervention letters to providers that explain this project, the nature of the problem, and identify a list of the provider's patients who have been contacted, as the provider and patient letters will be sent at approximately the same time; describe evidence and guidelines regarding oral anticoagulation
    - Delayed intervention letters to providers that explain this project, the nature of the problem, and identify a list of the provider's patients who are at risk for stroke and have not been treated with an oral anticoagulant; describe evidence and guidelines regarding oral anticoagulation
- Response mailer that gives the provider the opportunity to share the rationale for his or her patient(s) not being on OAC

- Web portal with access to clinical practice guidelines, decision support tools including from professional societies, podcasts, and case studies targeted at improving the appropriate use of OAC for AF
- Information sheet describing top misperceptions of barriers to OAC use
    1. Misperception of benefit and risk of aspirin: it is neither safe nor effective
    2. Misperception around risk of resuming OAC (months) after bleeding
    3. Misperception around risk of OAC regarding patients who fall
    4. Opportunities with NOACs for patients who have not tolerated warfarin
    5. Concern about lack of an antidote for the NOACs

IX. METHOD OF ASSIGNING PATIENTS TO AN INTERVENTION

An electronic program, developed by the central coordinating center (Harvard Pilgrim), will be used by the participating Data Partners to identify their eligible member-patients who meet the inclusion and exclusion criteria for this trial. The patient billing claims records will not be linked to pharmacy data until after randomization, so the inclusion criterion of no OAC medication fill during the 12 months prior to randomization will not be applied until after randomization. This will be done immediately for the early intervention group, and in the delayed intervention group, not until 12 months after at least 80% of early intervention mailing have occurred. Patients will be randomly assigned in a 1:1 ratio, via the program, to early patient-level and provider-level intervention versus delayed provider-level intervention. A list of eligible member-patients will stay at each Data Partner, and identifiable patient-level data will not be shared with the central coordinating center (Harvard Pilgrim) or the statistical analysis center (Duke Clinical Research Institute). The Data Partners will remove any individuals who cannot be included in research studies, which includes certain Centers for Medicare and Medicaid Services and Administrative Services Only patients. There are several other reasons that could prevent mailing of the intervention, to members and/or their providers (list may not be exhaustive):

- Member is now deceased
- Member disenrolls from health plan
- Member transitions to a plan that does not allow for inclusion in research
- Member has a recent anticoagulant dispensing
- Member has an incorrect/missing/bad address
- Member is added to a do-not-contact list at the health plan

The list of randomized individuals will include the provider on each patient's most recent encounter with an AF diagnosis as of the last date in the current approved data used for cohort identification. When the identified provider is an individual, this provider will receive the provider intervention materials. When the identified provider is a facility, Data Partners will select an alternate provider or conduct a member only mailing based on their health plan policies and the decision of the health plan's leadership. An alternative provider may be a patient's PCP or other recent clinician who provided care. At least one health plan intends to mail letters to the member only when an individual provider is not identified with the most recent AF diagnosis given the large volume of members.

As with the member-patient list, the provider list will be kept by each Data Partner and not shared, as each Data Partner will be mailing the intervention materials to their respective patients and providers.

X. ETHICAL CONSIDERATIONS

This study will be conducted, where appropriate, in accordance with good research practice as outlined by Good Clinical Practice (GCP) and the International Conference on Harmonisation (ICH) and in accordance with the ethical principles underlying the United States Code of Federal Regulations, Title 21, Part 50 (21CFR50). The study will be conducted in compliance with the protocol. The protocol and any amendments will receive Institutional Review Board/Independent Ethics Committee (IRB/IEC) approval/favorable opinion before initiation of the study. Study personnel involved in conducting this study will be qualified by education, training, and experience to perform their respective tasks. This study will not use the services of study personnel where sanctions have been invoked or where there has been scientific misconduct or fraud (e.g., loss of medical licensure; debarment).

Insurance companies do not ordinarily identify their members with AF who might be appropriate candidates for OACs, and they perform no standard outreach to either clinicians or members regarding treatment of AF. Individuals who are identified for this trial will have had their health care providers bill under the diagnosis code of AF at least twice, with at least one billed diagnosis within the last 12 months. Thus, this trial involves no new identification of AF, since the method of ascertainment is through billing by providers based on their diagnosing AF. The current standard of care is for clinicians and their patients with AF to decide together whether anticoagulation for the diagnosed atrial fibrillation is appropriate. All Data Partners collaborating in the IMPACT-AFib trial are insurers (or affiliates of insurers), not care providers, and the clinicians are not employees of the Data Partners.

There is no current evidence that the communications by insurers (like those being assessed in this trial) will have any effect on use of anticoagulation. All eligible patients included in the trial who do not appear to fill an OAC prescription will receive an intervention, as they will either be in the early intervention or the delayed intervention cohort.

XI. INSTITUTIONAL REVIEW BOARD/INDEPENDENT ETHICS COMMITTEE

Before study initiation, the co-principal investigators must have written and dated approval/favorable opinion from the IRB/IEC for the protocol, and any other written information to be provided to prospective participants.

The investigators should provide the IRB/IEC with reports, updates, and other information (e.g., expedited safety reports, amendments, and administrative letters) according to regulatory requirements or institution procedures.

XII. INFORMED CONSENT

Consent will be waived. The reasons for the waiver include:

1. The contacting of the delayed intervention patients for consent would be an intervention by itself and might affect the results of the trial.
2. The intervention is entirely consistent with a quality improvement initiative that the health plans could initiate on their own. Thus, the intervention is very low risk for harm.
3. The intervention only adds on to the existing care of and programs for patients. There are no restrictions placed on the delayed intervention group, as a result of the trial.
4. It would be impractical to collect informed consent on the patients included in this trial.

The waiver of informed consent is consistent with the approach that has been taken in several similar clinical trials, the first using individual patient randomization, the others cluster randomized:

1. UH3 Pragmatic Trial of Population-based Programs to Prevent Suicide Attempt (NCT02326883)
2. The HMO Research Network CERT: A randomized trial of direct-to-patient communication to enhance adherence to beta-blocker therapy following myocardial infarction (NCT00211172)
3. MI FREE: A Randomized Evaluation of First-dollar Coverage for Post-MI Secondary Prevention Therapies (NCT00566774)
4. STOPCRC: Strategies and Opportunities to Stop Colon Cancer in Priority Populations (NCT01742065)

XIII. INDEPENDENT ADVISORY COMMITTEE

An independent advisory committee (IAC), consisting of members independent from the study team and with expertise in statistics, anticoagulation for atrial fibrillation, engagement, health plan management, and/or in practical clinical trials, will serve to enhance patient safety and trial integrity. The IAC will meet with study leadership at least twice. The first meeting will occur before intervention materials are mailed, and the second will take place two months after all mailings are sent. In addition, other meetings will occur if deemed necessary by the IAC chair, study leads and/or the FDA. The study team will provide the IAC with the study protocol before any intervention materials are mailed and will brief the IAC on any issues (or lack thereof) that have been raised 2 months after the last mailing has occurred. The IAC will advise on concerns that arise. An IAC charter describes the roles, responsibilities, and operations of the IAC.

XIV. STATISTICAL CONSIDERATIONS

A. SAMPLE SIZE AND POWER CALCULATIONS

Our target sample size is 80,000 patients. All enrolled patients and/or their providers will be targeted by mailing (early or delayed) provided they are eligible at time of mailing and able to be contacted.

1. Primary Endpoint

The following assumptions were used to determine the sample size and power for the primary endpoint assessing the proportion of AF patients with evidence of at least one OAC prescription fill through the date on which at least 80% of eligible study participants have at least 12 months of follow-up time:

- 33% OAC initiation rate in the delayed intervention arm over the first year of the study
  - In a Sentinel feasibility assessment of identified health plan members meeting similar inclusion and exclusion criteria as this trial, 33% had evidence of OAC initiation in a 1 year period of follow-up.
- 38% OAC initiation rate in the early intervention arm (a 5% absolute improvement in OAC initiation over the 33% OA initiation expected in thedelayed intervention arm over 1-year follow-up)
- 1-year attrition rate: 30% dropout or lost-to-follow-up
- Two-sided type I error of 0.05
- Roughly 10,000 patients (Table below) will yield more than 99% power to detect a 5% absolute difference

Power	Total sample size (2-arm)	Early Intervention	Delayed Intervention
90%	5610	2805	2805
95%	6910	3455	3455
99%	9718	4859	4859

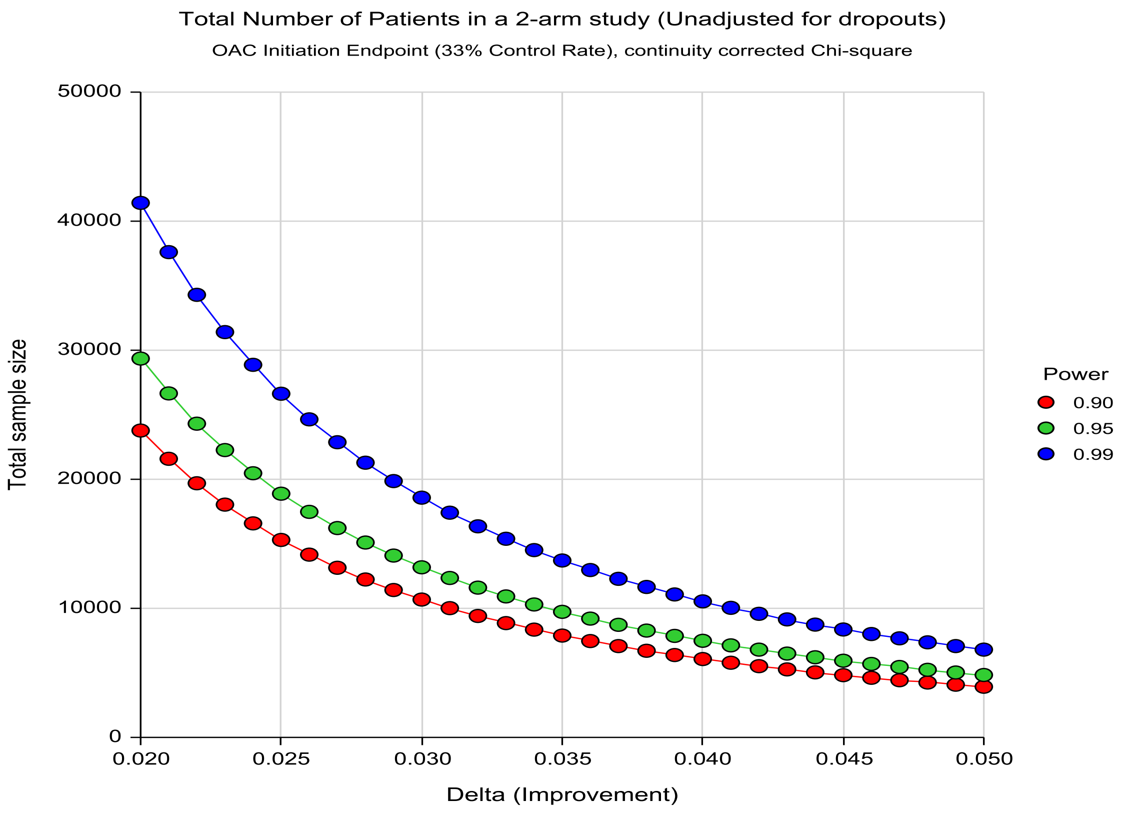

2. Important Secondary Analysis of Stroke or TIA

A study with approximately 80,000 patients is well powered for the outcome of stroke or TIA over the course of the follow-up through the date on which at least 80% of eligible study participants have at least 12 months of follow-up time, under certain assumptions listed below:

- 1-year stroke or TIA rate: 18% among patients not treated with OAC
- 1-year stroke or TIA rate: 7% among patients treated with at least 1 OAC fill
- Duration of follow-up: 1 year
- 33% of delayed intervention patients will have at least 1 fill of OAC, meaning the 1 year stroke or TIA rate in the delayed intervention group would be 14.4%
- If 38% of early intervention patients have at least 1 fill of OAC (meaning the 1 year stroke or TIA rate in the early intervention group would be 13.82%, i.e., an absolute reduction of 0.55%), the study will have 46% power to detect this 0.55% reduction. However, if 40.5% of early intervention patients have at least 1 fill of OAC (meaning the 1 year stroke or TIA rate in the early intervention group would be 13.54%, i.e., an absolute reduction of 0.83%), the study will have 80% power to detect this 0.83% reduction.

- 1-year attrition rate: 30% dropout or lost-to-follow-up
- Two-sided type I error of 0.05
- The sample size has 80% power to detect a 0.5% absolute reduction in stroke, assuming a cumulative 1-year incidence of stroke of 4.2% in control (delayed intervention arm) patients and 3.7% in intervention (early intervention arm) patients. The assumption is that patients not on oral anticoagulation have an annual stroke rate of 5%, and stroke will be reduced by 50% (HR=0.5) in the treated (anticoagulated) population. The 80% power requires that 52% of early intervention arm patients are treated at 1-year as compared to 33% in the control arm.

B. POPULATIONS FOR ANALYSIS

All analyses will be based on a modified intention-to-treat principle (i.e., all identified early intervention patients who meet eligibility and are mailed the intervention will be included; using "pseudo" wave assignments, all identified delayed intervention patients who meet eligibility at the time of corresponding early mailings will be included in the analysis). The primary endpoint will be calculated based on the proportion of patients with evidence of at least one OAC prescription fill over the course of the follow-up through the date on which at least 80% of eligible study participants have at least 12 months of follow-up time. An as-randomized analysis will be performed for the primary endpoint as a sensitivity analysis. All possible person-time will be used to assess participants' outcomes; patients will have different duration of follow-up and that will be accounted for in the analyses. Patients will be censored from the analysis at the time of death, disenrollment from the health plan, loss of medical or pharmacy coverage, or change in eligibility for inclusion in research based on health plan membership. The statistical analysis plan (SAP) provided more information and outlines in detail how granular aggregate data will be received from each Data Partner in lieu of patient-level data.

XV. OUTCOME DEFINITIONS

All outcome definitions will be based on available claims data.

1. Evidence of OAC start: the proportion of AF patients who have at least 4 INR values measured or who fill at least one OAC prescription for apixaban, dabigatran, edoxaban, rivaroxaban, or warfarin over the course of the follow-up through the date on which at least 80% of eligible study participants have at least 12 months of follow-up time
2. Time to OAC start: the time to first OAC initiation, as defined by the first fill date for apixaban, dabigatran, edoxaban, rivaroxaban, or warfarin. If there was no prescription fill for these medications, but the patient had 4 or more INR values measured over the study period, the date of the first INR measurement will be used for initiation of OAC.
3. OAC prescription coverage: the proportion of days that AF patients have OAC prescription fills over the duration of the study. The assumption is that a 30-day or 90-day supply will last for the planned period, even in the case of warfarin, when the length of time that a prescription lasts may be less well defined. Patients will only be included in this secondary analysis, if they had a prescription fill for apixaban, dabigatran, edoxaban, rivaroxaban, or warfarin.
4. OAC rates at follow-up: proportion of patients with an active oral anticoagulant dispensing at the end of the one-year follow-up. This will include patients with apixaban, dabigatran, edoxaban, rivaroxaban, or warfarin medication fills that encompass the end of the study period. If there was no prescription fill for these medications, this will also include patients with 4 or

more INR values measured over the study period, who had an INR value measured within 60 days of the end of the approximately one-year follow-up.

5. Rate of stroke or TIA: the incident rate of stroke or TIA related hospitalizations; stroke, excluding transient ischemic attacks (TIAs), will also be assessed
6. Rate of bleeding related hospitalization: the incident rate of hospitalizations for any bleeding
7. Health care utilization: the total counts of health care utilization (number of outpatient visits, emergency department visits, hospital admissions, etc.) for AF patients, including AF and non-AF related care
8. In-patient all-cause mortality: patients with in-hospital death
9. All-cause mortality: in a subset of patients with mortality status

XVI. STATISTICAL ANALYSES

A. DEMOGRAPHICS AND BASELINE CHARACTERISTICS

Baseline characteristics of eligible early and delayed intervention cohorts will be based on the claims data at the time of randomization. Frequency distribution and summary statistics for demographic and baseline variables will be presented by early intervention group, delayed intervention group, and for the overall study population. Key demographic and baseline variables to be summarized include: geographic region, age, sex, risk factors for stroke, risk factors for bleeding, and select comorbid conditions. Continuous variables will be summarized as mean (±SD) and median (25th, 75th percentiles); the comparison between the two groups will be conducted using Wilcoxon rank-sum test. For the continuous variables in combined study population, the mean will be summarized. Categorical variables will be presented as counts (percentages) and will be compared between groups by using Pearson's chi-square or Fisher's exact test, as appropriate.

B. RESPONSES FROM PROVIDERS

As part of the provider intervention materials, providers will have the opportunity to respond to the patient's health plan and give an explanation for why their patients were not being treated with OAC via a postage paid postcard. The data collected from these responses will be aggregated by rationale for non-treatment and reported as counts (percentages) by the coordinating center. Data will not be linked to specific patients or providers.

C. EFFECTIVENESS ANALYSES

The proportion of patients with evidence of at least one OAC medication fill over the course of the follow-up, the primary endpoint, will be summarized and compared between the early intervention and delayed intervention arms. The definition for OAC medication fill will be an OAC medication billing in the pharmacy claims or at least 4 INR tests billed in the laboratory claims (indicative of OAC use that was not billed through the pharmacy claims data). To evaluate whether the comprehensive evaluation and customized multilevel (patient-level and provider-level) educational interventions increases the rate of use of oral anticoagulants at one year post intervention, we will evaluate the odds ratio (i.e. the odds of an average patient in the early intervention group taking OAC at 1 year compared to the odds of an average patient in the delayed intervention group taking OAC at 1 year) using logistic regression to account for the effect of Data Ppartner and adjust for other potential baseline risk factors.[38] We will

present the odds ratio, 95% confidence interval, and p-value to show whether there is a difference in rate of use of OAC at one year post intervention between the early intervention and delayed intervention groups. Similarly, we will analyze the proportion of days covered with an OAC prescription fill over the course of the follow-up. Patients will only be included in this analysis, if they had a prescription fill for apixaban, dabigatran, edoxaban, rivaroxaban, or warfarin.

Event rates for the following secondary outcomes will be summarized by treatment group, as defined by claims data:

- Stroke or TIA
- Ischemic stroke or unknown stroke
- Hemorrhagic stroke
- Hospitalization for any bleeding
- Composite of ischemic or hemorrhagic stroke
- Composite of ischemic stroke, hemorrhagic stroke, and hospitalization for any bleeding

For time-to-event outcomes, Kaplan-Meier cumulative incidence estimates and log-rank test will be utilized.[39] In addition, Cox proportional hazards model with early intervention vs. delayed intervention as main effect will be used to assess the clinical outcomes, after adjusting for baseline risk factors.[40] A robust sandwich covariance estimate or a frailty model will be used to account for effect of Data Partner. The hazard ratio, 95% confidence interval and p-value will be presented to summarize the difference in the risk of clinical outcome between early intervention and delayed intervention group. In-hospital death or medically attended death will be collected through claims data. Analyses will be performed using SAS software version 9.4 or higher (SAS Institute, Inc., Cary, NC).

More details on the statistical methods and analyses will be provided in the SAP. The SAP will be finalized prior to study database lock, 12 months after at least 80% of the early intervention mailings. Of note, all patient-level data will be maintained by the Data Partners, so analyses are expected to be conducted via a distributed SAS programming code developed by the coordinating center and results are expected to be returned, by Data Partner, in aggregate to the coordinating center. Analyses will be examined by Data Partner and across Data Partners.

XVII. DISSEMINATION PLAN OVERVIEW

A manuscript describing the study design will be published. A manuscript will also be written describing the results on the primary and secondary endpoints based on the date on which at least 80% of eligible study participants have at least 12 months of follow-up time, and separately once at least 80% of members have at least 24 months of follow-up time (if 24-month aanalysis is conducted). Finally, Data Partner-specific results will be shared with each Data Partner at both time points.

XVIII. REFERENCES

1. Wann LS, Curtis AB, January CT, et al. 2011 ACCF/AHA/HRS focused update on the management of patients with atrial fibrillation (Updating the 2006 Guideline): a report of the American College of Cardiology Foundation/American Heart Association Task Force on Practice Guidelines. *J Am Coll Cardiol.* 2011;57(2):223-242.

2. Feinberg WM, Blackshear JL, Laupacis A, Kronmal R, Hart RG. Prevalence, age distribution, and gender of patients with atrial fibrillation: analysis and implications. *Archives of internal medicine.* 1995;155(5):469.

3. Novaro GM, Asher CR, Bhatt DL, et al. Meta-analysis comparing reported frequency of atrial fibrillation after acute coronary syndromes in Asians versus whites. *The American journal of cardiology.* 2008;101(4):506-509.

4. Go AS, Hylek EM, Phillips KA, et al. Prevalence of diagnosed atrial fibrillation in adults. *JAMA: the journal of the American Medical Association.* 2001;285(18):2370-2375.

5. Furberg CD, Psaty BM, Manolio TA. Prevalence of atrial fibrillation in elderly subjects (the Cardiovascular Health Study). *The American journal of cardiology.* 1994;74(3):236-241.

6. Psaty BM, Manolio TA, Kuller LH, et al. Incidence of and risk factors for atrial fibrillation in older adults. *Circulation.* 1997;96(7):2455-2461.

7. Miyasaka Y, Barnes ME, Gersh BJ, et al. Secular trends in incidence of atrial fibrillation in Olmsted County, Minnesota, 1980 to 2000, and implications on the projections for future prevalence. *Circulation.* 2006;114(2):119-125.

8. Benjamin EJ, Levy D, Vaziri SM, D'Agostino RB, Belanger AJ, Wolf PA. Independent risk factors for atrial fibrillation in a population-based cohort. *JAMA: the journal of the American Medical Association.* 1994;271(11):840-844.

9. Wolf PA, Abbott RD, Kannel WB. Atrial fibrillation: a major contributor to stroke in the elderly: the Framingham Study. *Archives of internal medicine.* 1987;147(9):1561.

10. Wolf PA, Dawber TR, Thomas Jr HE, Kannel WB. Epidemiologic assessment of chronic atrial fibrillation and risk of stroke. *Neurology.* 1978;28(10):973-973.

11. Flegel K, Shipley M, Rose G. Risk of stroke in non-rheumatic atrial fibrillation. *The Lancet.* 1987;329(8532):526-529.

12. Hart RG, Pearce LA. Current status of stroke risk stratification in patients with atrial fibrillation. *Stroke.* 2009;40(7):2607-2610.

13. Lin HJ, Wolf PA, Kelly-Hayes M, et al. Stroke severity in atrial fibrillation: the Framingham study. *Stroke.* 1996;27(10):1760-1764.

14. Miyasaka Y, Barnes ME, Bailey KR, et al. Mortality trends in patients diagnosed with first atrial fibrillation: a 21-year community-based study. *J Am Coll Cardiol.* 2007;49(9):986-992.

15. Wilke T, Groth A, Mueller S, et al. Oral anticoagulation use by patients with atrial fibrillation in Germany. Adherence to guidelines, causes of anticoagulation under-use and its clinical outcomes, based on claims-data of 183,448 patients. *Thromb Haemost.* 2012;107(6):1053-1065.

16. Connolly SJ, Pogue J, Eikelboom J, et al. Benefit of oral anticoagulant over antiplatelet therapy in atrial fibrillation depends on the quality of international normalized ratio control achieved by

centers and countries as measured by time in therapeutic range. *Circulation.* 2008;118(20):2029-2037.

17. Granger CB, Alexander JH, McMurray JJ, et al. Apixaban versus warfarin in patients with atrial fibrillation. *N Engl J Med.* 2011;365(11):981-992.

18. Patel MR, Mahaffey KW, Garg J, et al. Rivaroxaban versus warfarin in nonvalvular atrial fibrillation. *N Engl J Med.* 2011;365(10):883-891.

19. Connolly SJ, Ezekowitz MD, Yusuf S, et al. Dabigatran versus warfarin in patients with atrial fibrillation. *N Engl J Med.* 2009;361(12):1139-1151.

20. Giugliano RP, Ruff CT, Braunwald E, et al. Edoxaban versus Warfarin in Patients with Atrial Fibrillation. *N Engl J Med.* 2013.

21. Gage BF, Waterman AD, Shannon W, Boechler M, Rich MW, Radford MJ. Validation of clinical classification schemes for predicting stroke: results from the National Registry of Atrial Fibrillation. *JAMA.* 2001;285(22):2864-2870.

22. January CT, Wann LS, Alpert JS, et al. 2014 AHA/ACC/HRS guideline for the management of patients with atrial fibrillation: a report of the American College of Cardiology/American Heart Association Task Force on Practice Guidelines and the Heart Rhythm Society. *J Am Coll Cardiol.* 2014;64(21):e1-76.

23. Hsu JC, Maddox TM, Kennedy KF, et al. Oral Anticoagulant Therapy Prescription in Patients With Atrial Fibrillation Across the Spectrum of Stroke Risk: Insights From the NCDR PINNACLE Registry. *JAMA Cardiol.* 2016;1(1):55-62.

24. Hess CN, Broderick S, Piccini JP, et al. Antithrombotic therapy for atrial fibrillation and coronary artery disease in older patients. *Am Heart J.* 2012;164(4):607-615.

25. Partington SL, Abid S, Teo K, Oczkowski W, O'Donnell MJ. Pre-admission warfarin use in patients with acute ischemic stroke and atrial fibrillation: The appropriate use and barriers to oral anticoagulant therapy. *Thrombosis research.* 2007;120(5):663-669.

26. Go AS, Hylek EM, Borowsky LH, Phillips KA, Selby JV, Singer DE. Warfarin use among ambulatory patients with nonvalvular atrial fibrillation: the anticoagulation and risk factors in atrial fibrillation (ATRIA) study. *Annals of internal medicine.* 1999;131(12):927-934.

27. Waldo AL, Becker RC, Tapson VF, Colgan KJ, Committee NS. Hospitalized patients with atrial fibrillation and a high risk of stroke are not being provided with adequate anticoagulation. *J Am Coll Cardiol.* 2005;46(9):1729-1736.

28. Birman-Deych E, Radford MJ, Nilasena DS, Gage BF. Use and effectiveness of warfarin in Medicare beneficiaries with atrial fibrillation. *Stroke.* 2006;37(4):1070-1074.

29. Walker AM, Bennett D. Epidemiology and outcomes in patients with atrial fibrillation in the United States. *Heart Rhythm.* 2008;5(10):1365-1372.

30. Nieuwlaat R, Capucci A, Lip GY, et al. Antithrombotic treatment in real-life atrial fibrillation patients: a report from the Euro Heart Survey on Atrial Fibrillation. *Eur Heart J.* 2006;27(24):3018-3026.

31. Connolly SJ, Eikelboom J, Joyner C, et al. Apixaban in patients with atrial fibrillation. *N Engl J Med.* 2011;364(9):806-817.

32. WorldHealthOrganization(WHO).Adherenceto Long-term Therapies: Evidence for Action. WHO website. http://www.who.int/chp/knowledge /publications/adherence_report/en/index.html. Accessed November 21.

33. Frishman WH. Importance of medication adherence in cardiovascular disease and the value of once-daily treatment regimens. *Cardiology in review.* 2007;15(5):257-263.

34. Brown TM, Siu K, Walker D, Pladevall-Vila M, Sander S, Mordin M. Development of a conceptual model of adherence to oral anticoagulants to reduce risk of stroke in patients with atrial fibrillation. *Journal of managed care pharmacy : JMCP.* 2012;18(5):351-362.

35. Costa E, Giardini A, Savin M, et al. Interventional tools to improve medication adherence: review of literature. *Patient preference and adherence.* 2015;9:1303-1314.

36. Osterberg L, Blaschke T. Adherence to medication. *N Engl J Med.* 2005;353(5):487-497.

37. Chen R, Valladares C, Corbal I, Anani N, Koch S. Early Experiences from a guideline-based computerized clinical decision support for stroke prevention in atrial fibrillation. *Studies in health technology and informatics.* 2013;192:244-247.

38. Liang KY, Zeger SL. Longitudinal Data Analysis Using Generalized Linear Models. *Biometriks.* 1986;73:13-22.

39. Kaplan EL, Meier P. Nonparametric estimation from incomplete observations. *J Am Statist Assoc.* 1958;53:457-481.

40. Cox DR. Regression models and life-tables. *J Royal Statist Soc B.* 1972;34:187-220.

FDA-CATALYST STATISTICAL ANALYSIS PLAN

IMPLEMENTATION OF A RANDOMIZED CONTROLLED TRIAL TO IMPROVE TREATMENT WITH ORAL ANTICOAGULANTS IN PATIENTS WITH ATRIAL FIBRILLATION (IMPACT-AFib)

Prepared by: Wensheng Vincent He, PhD[1]; Hussein R. Al-Khalidi, PhD[1]; Sengwee Toh, ScD[2]; and Noelle Cocoros, DSc, MPH[2] on behalf of the IMPACT-AFib Workgroup

Author Affiliations: 1. Duke Clinical Research Institute, Duke University Medical Center, Durham, NC; 2. Department of Population Medicine, Harvard Pilgrim Health Care Institute and Harvard Medical School, Boston, MA

Version 1.0

March 7, 2019

The Sentinel System is sponsored by the U.S. Food and Drug Administration (FDA) to proactively monitor the safety of FDA-regulated medical products and complements other existing FDA safety surveillance capabilities. The Sentinel System is one piece of FDA's Sentinel Initiative, a long-term, multi-faceted effort to develop a national electronic system. Sentinel Collaborators include Data and Academic Partners that provide access to healthcare data and ongoing scientific, technical, methodological, and organizational expertise. The Sentinel Coordinating Center is funded by the FDA through the Department of Health and Human Services (HHS) Contract number HHSF223201400030I.

FDA-Catalyst Statistical Analysis Plan

Implementation of a Randomized Controlled Trial to Improve Treatment with Oral Anticoagulants in Patients with Atrial Fibrillation (IMPACT-AFib)

Table of Contents

I. OVERVIEW 1
- A. Primary Endpoint 1
- B. Secondary Endpoints 1
- C. Exploratory Endpoints 1
- D. Data Source 2
- E. Patient Inclusion Criteria 2
- F. Study Design and Duration 4
- G. Sample Size Justification 8
  - *1. Primary Endpoint 8*
  - *2. Important Secondary Outcome of Stroke or TIA 9*

II. STATISTICAL ANALYSIS 9
- A. Methods for Analysis in Distributed Data Networks 12
- B. Demographics and Baseline Characteristics 12
- C. Primary Endpoint Data Analyses 13
  - *1. Adjusted Analysis Model 13*
  - *2. Analysis Results Interpretation 13*
- D. Secondary Endpoints Data Analyses 14
  - *1. OAC Initiation 14*
  - *2. OAC Adherence 14*
  - *3. Proportion of Patients Actively on OAC at the End of Follow-up 15*
  - *4. Clinical Outcomes 15*
  - *5. Health Care Utilization 16*

III. REFERENCES 17

IV. APPENDIX 18
- A. Tables 18
- B. CONSORT 2010 Worksheet for Randomized Trials 23
- C. ICD-10 Codes for Defining Clinical Outcomes 25
- D. Analysis methods for distributed networks 27
  - *1. Meta-analysis 27*
  - *2. Case-centered Logistic Regression 27*
  - *3. Distributed Regression 27*

I. OVERVIEW

This is the statistical analysis plan (SAP) for **IM**plementation of a randomized controlled trial to im**P**rove treatment with oral **A**nti**C**oagulan**T**s in patients with **A**trial **F**ibrillation (IMPACT-AFib) study. The purpose of this document is to provide an overview of the study design and study objectives, outline the types of analyses and data presentations relevant to the study objectives, and to provide a detailed description of the methods in which the statistical analyses will be conducted to meet protocol objectives. This plan is a supplement to the materials provided in the IMPACT-AFib protocol. This SAP does not contain all the protocol details and is intended to be read in conjunction with the full protocol. Only analytic decisions are documented here.

A. PRIMARY ENDPOINT

IMPACT-AFib is a prospective randomized controlled clinical trial that will evaluate whether a patient and provider education intervention increases the proportion of patients with atrial fibrillation (AF) who fill at least one oral anticoagulant (OAC) over the course of follow-up. Follow up is through the date on which at least 80% of eligible study participants have at least 12 months of follow-up time. As described in the protocol, there is an "early intervention" arm (who receive patient and provider mailings) and a "delayed intervention" arm (who receive provider mailings only, ~12 months after the early intervention mailings occur).

B. SECONDARY ENDPOINTS

We will evaluate the impact of the patient and provider education intervention on the endpoints listed below at the end of follow-up (i.e., the date on which at least 80% of eligible study participants have at least 12 months of follow-up time):

- The incidence rate of stroke or transient ischemic attack (TIA) hospitalizations
- The incidence rate of stroke hospitalizations
- The time to first OAC prescription fill
- The proportion of days covered by OAC prescription fills
- Proportion of patients actively on OAC at 12 months of follow-up
- The incidence rate of hospitalization for any bleeding
- All-cause in-hospital mortality rates
- All-cause mortality rates among patients with accurate out-of-hospital mortality data, if data are available (such as Medicare Advantage patients)
- Health care utilization, reported as counts of number of health care utilization events (outpatient visits, days hospitalized, number of emergency department visits, etc.)

Depending on the review of preliminary data, the primary endpoint and some secondary endpoints may be examined separately by warfarin and novel OAC. This will be descriptive only and will not include formal statistical testing.

C. EXPLORATORY ENDPOINTS

We will evaluate the effect of the early and delayed education interventions on the primary and secondary endpoints once at least 80% of eligible study participants have at least 24 months of follow-up time (the early intervention includes mailing to the patient and provider while the delayed intervention, at ~12 months follow-up, only includes a provider mailing). Note that we may not conduct

these analyses if the results of the primary outcome are null in the earlier assessment. Therefore, this statistical analysis plan does not include the details of analyses for a 24 months assessment.

D. DATA SOURCE

The data used for the study are claims data from the five participating sites, transformed into the Sentinel Common Data Model. At the time of analysis, the data available in the Sentinel Distributed Database (i.e., those approved and in use for Sentinel routine surveillance activities) will be used to assess the primary and secondary endpoints.

As background, the identification and creation of the study cohort was based on the claims data in the Sentinel Distributed Database plus linked “fresh” data (i.e., about 1 month old) for pharmacy claims and enrollment information. The target population for the study was those members enrolled in the sites who did not have evidence of an OAC medication dispensing in the 12 months prior to randomization; the fresh and production data ensured we identified those eligible for the trial. The data used for routine Sentinel activities are several months old – hence the need for certain “fresh” data – and the date of the last available claims varies by site.

E. PATIENT INCLUSION CRITERIA

All inclusion and exclusion criteria were determined by claims data. For entry into the study, the following criteria MUST be met at the date of randomization:

1. Two or more diagnoses of AF (ICD-10-CM codes I48.0, 148.1, 148.2, 148.4, or I48.91; ICD-9-CM codes 427.3 or 427.31) at least one day apart and with at least one diagnosis within the last 12 months prior to the last date in the current approved data used for cohort identification
2. CHA_2DS_2-VASc score of 2 or greater at the time of the randomization (i.e., as of the last date in the current approved data used for cohort identification). The ICD-9/10-CM coding for CHA_2DS_2-VASc is shown in Table 1. The complete code list for inclusion and exclusion criteria is accessible on the Sentinel website (https://www.sentinelinitiative.org/FDA-catalyst/projects/implementation-randomized-controlled-trial-improve-treatment-oral-anticoagulants-patients).
3. Medical and pharmacy insurance coverage as identified via administrative claims data as of the date of randomization
4. Age 30 years or greater as of the last date in the current approved data used for cohort identification

Table 1. Coding for CHA2DS2-VASc components

	Component	Codes
C	Congestive heart failure (or left ventricular systolic dysfunction)	**ICD-10-CM:** I09.81, I11.0, I13.0, I13.2, I42.0, I42.5-I42.9, I43, I50.1, I50.20-23, I50.30–I50.33, I50.40-43; I50.9; **ICD-9-CM:** 398.91, 402.01, 402.11, 402.91, 404.01, 404.03, 404.11, 404.13, 404.91, 404.93, 425.2, 425.4, 425.5, 425.7, 425.9, 428.0-428.4, 428.20-428.23, 428.30-428.33, 428.40-428.43, 428.9
H	Hypertension: blood pressure consistently above 140/90 mmHg (or treated hypertension on medication)	**ICD-10-CM:** I10–I16 and subcodes, I67.4, N26.6; **ICD-9-CM:** 401-405, 401.0, 401.1, 401.9, 402.0, 402.1, 402.9, 403.0, 403.1, 403.9, 404.0, 404.1, 404.9, 405.0, 405.1, 405.9, 437.2, 402.**, 403.**, 404.**, 405.**; **CPT:** 4050F
A_2	Age ≥75 years	
D	Diabetes Mellitus	**ICD-10-CM:** E08.31- E08.36, E08.3**, E08.40, E08.42, E08.51, E08.52, E08.59, E08.65, E09.31-E09.36, E09.3**, E09.40, E09.42, E09.51, E09.52, E09.59, E10.1–E13.9 and subcodes; **ICD-9-CM:** 250.*, 357.2, 362.0, 249.7*, 250.**, 362.0*, 366.41
S_2	Prior stroke or TIA or thromboembolism	**ICD-10-CM:** I60.**, I60.2, I60.4, I60.6-I60.9, I61.*, I62.9, I63.0*, I63.0**, I63.1*, I63.1**, I63.2*, I63.2**, I63.3*, I63.3**, I63.4*, I63.4**, I63.5*, I63.5**, I63.6, 163.8, I63.9, I69.00, I69.0**, I69.10, I69.1**, I69.30, I69.3**, I69.8** except I69.898, I69.9** except I69.998, S06.34, S06.34*A, and S063.34*D, S06.35, S06.35*A, and S063.35*D, S06.36, S06.36*A, and S063.36*D, S06.6, S06.6X*A, S06.6X*D, Z86.73, H34.00, H34.219, H34.239, H34.9, I67.82, I74.**, I74.2-I74.9, K76.3, N28.0, T81.718A, G45.8, G45.9; **ICD-9-CDM:** 438.0-438.8, 852.0, 853.0, 433.*1, 434.*1, 438.1*, 438.2*, 438.3*, 438.4*, 438.5*, 438.81-428.85, 852.0*, 853.0*, V12.54, 444, 444.*, 444.**, 453.9, 573.4, 362.30-362.34, 434.00, 435, 435.8, 435.9; **CPT:** 34101, 34111, 34201, 34203
V	Vascular disease (e.g. peripheral artery disease, myocardial infarction, aortic plaque)	**ICD-10-CM**[1]**:** E08.51, E08.52, E09.51, E09.52, E10.51, E10.52, E11.51, E11.52, E13.51, E13.52, I21.0*, I21.1*, I21.2*, I21.3, I21.4, I22.*, I25.7, I25.70-I25.73, I25.70*, I25.71*, I25.72*, I25.73*, I25.79, I25.79*, I25.810, I73.8, I73.9, I77.1, T82.2, T82.21 and subcodes, Z95.820, Z95.82
A	Age 65–74 years	
Sc	Sex category (i.e. female sex)	

[1] Only ICD-10-CM codes are presented for the vascular disease component given size of the list; complete code list, which includes ICD-9-CM, ICD-10-PCS, ICD-9-PCS, CPT, and HCPCS codes, is available on the Sentinel website.

Patients are excluded if they meet any of the following criteria:

1. Evidence of OAC medication fill during the 12 months prior to randomization (the delayed intervention group's treatment status will be assessed at the end of the 12 month follow-up period)
2. Conditions other than AF that require anticoagulation such as ever having mechanical prosthetic valve, deep venous thrombosis, or pulmonary embolism prior to the last date in the current approved data used for cohort identification ("ever" is operationalized as -6000 days from the index AF code)
3. Pregnancy identified within 6 months of the last date in the current approved data used for cohort identification
4. Any known history of intracranial hemorrhage prior to the last date in the current approved data used for cohort identification
5. Hospitalization for any bleeding within the last 6 months of the last date in the current approved data used for cohort identification
6. Patients with recent P2Y12 antagonist use (i.e., clopidogrel, prasugrel, or ticagrelor within 90 days of prior to randomization)

F. STUDY DESIGN AND DURATION

As described in detail in the protocol, the study is a prospective, randomized, and open-label educational intervention trial. Patients with AF and a CHA_2DS_2-VASc score of 2 or greater were randomized in a 1:1 ratio to an early intervention cohort and a delayed intervention cohort within each participating health plan. The definition for OAC medication fill was an OAC medication dispensing or at least 4 INR tests in the claims data.[2] The claims records of the patients randomized to the early intervention cohort were then linked to "fresh" (i.e., about 1 month old) pharmacy claims data at the time of randomization. Patients without evidence of an OAC medication fill during the 12 months prior to randomization were included in the patient-level and provider-level early educational intervention (patients randomized to this early intervention with evidence of an OAC medication fill during the 12 months prior to randomization were excluded from the trial). In addition to usual care, these patients and their providers received a one-time mailing at trial start. There were two waves of mailing for the early intervention cohort at most sites due to the practical challenges of claims data: the patients were assigned to wave 1 if they had a provider easily identified in the data (i.e., the provider associated with the most recent AF diagnosis is indeed an individual provider), and they were assigned to wave 2 if it was difficult to identify a provider (e.g., the first identified provider is actually a facility). Follow-up time started on the date of the respective wave 1 and wave 2 mailings for the early intervention patients.

The delayed intervention cohort will have received usual care over the initial study period. After the date on which at least 80% of all eligible study participants have at least 12 months of follow-up time, the treatment status of the delayed intervention group will be assessed via the Sentinel data available at that time, in addition to "fresh" pharmacy claims data. The providers of patients in the delayed cohort who did not receive OAC medication during the course of follow-up and still meet all inclusion criteria

[2]Not all OAC dispensings are captured in pharmacy claims, particularly, for warfarin due to some patients paying for medication out of pocket. INR tests are assumed indicative of OAC fills that were not billed through the claims. Four INR tests or values within a 12-month period will be used as a proxy since that is roughly the number of tests administered in the process of stabilizing dose.

will receive the provider-only education intervention (patients will not receive the educational materials unless no provider can be identified for a mailing).

Details on the analyses are provided in Section II. Here we describe the data sources for the **modified intention-to-treat** (primary) and **as-randomized** (sensitivity) analyses. Similar to early intervention cohort, the patients in the delayed intervention cohort will be assigned to wave 1 or wave 2 ("pseudo" wave assignments) depending on the difficulty in identifying the patient's provider (the intent is to handle them the same way as was done for the early intervention arm, for the modified intention-to-treat analysis). The follow-up for the delayed intervention patients will start on the date the wave 1 or 2 mailings took place for a given Data Partner's early intervention cohort. For both early and delayed intervention cohorts, any patients who die, are disenrolled, or get started on OAC between the randomization and early intervention mailing will be excluded from the analysis at each Data Partner. For both the early and delayed arms, exclusion criteria that are based on member medical history were assessed at the time of randomization. For the early intervention cohort, enrollment and treatment status were assessed at randomization for all and re-assessed at the wave 2 time point at some of the sites (this was at site discretion, in response to the lag between wave 1 and 2 mailings). The enrollment and treatment status of the delayed intervention cohort will be examined for eligibility at the same time point as the early intervention patients, meaning at the time of mailing per Data Partner, via the locked data (using the wave 1 and 2 dates).

Because the Sentinel Distributed Database will be used for analyses, and this information is refreshed approximately quarterly on different timetables for the different health plans, it is likely that when the required follow-up time is available for at least 80% of patients, there will be more than 12 months of follow-up for over 80% of patients. All participants' outcomes will be assessed using all possible person-time; patients will have different duration of follow-up and that will be accounted for in the analyses. Note that if the 24 month analysis is conducted, we will do that when at least 80% of members have at least 24 months of follow-up time.

A schematic diagram below shows the design of the early intervention period of the study: over the course of the follow-up, through the date on which at least 80% of eligible study participants have at least 12 months of follow-up time (see the protocol for the full study design and details):

Figure 1. Design of the early intervention period portion of the study

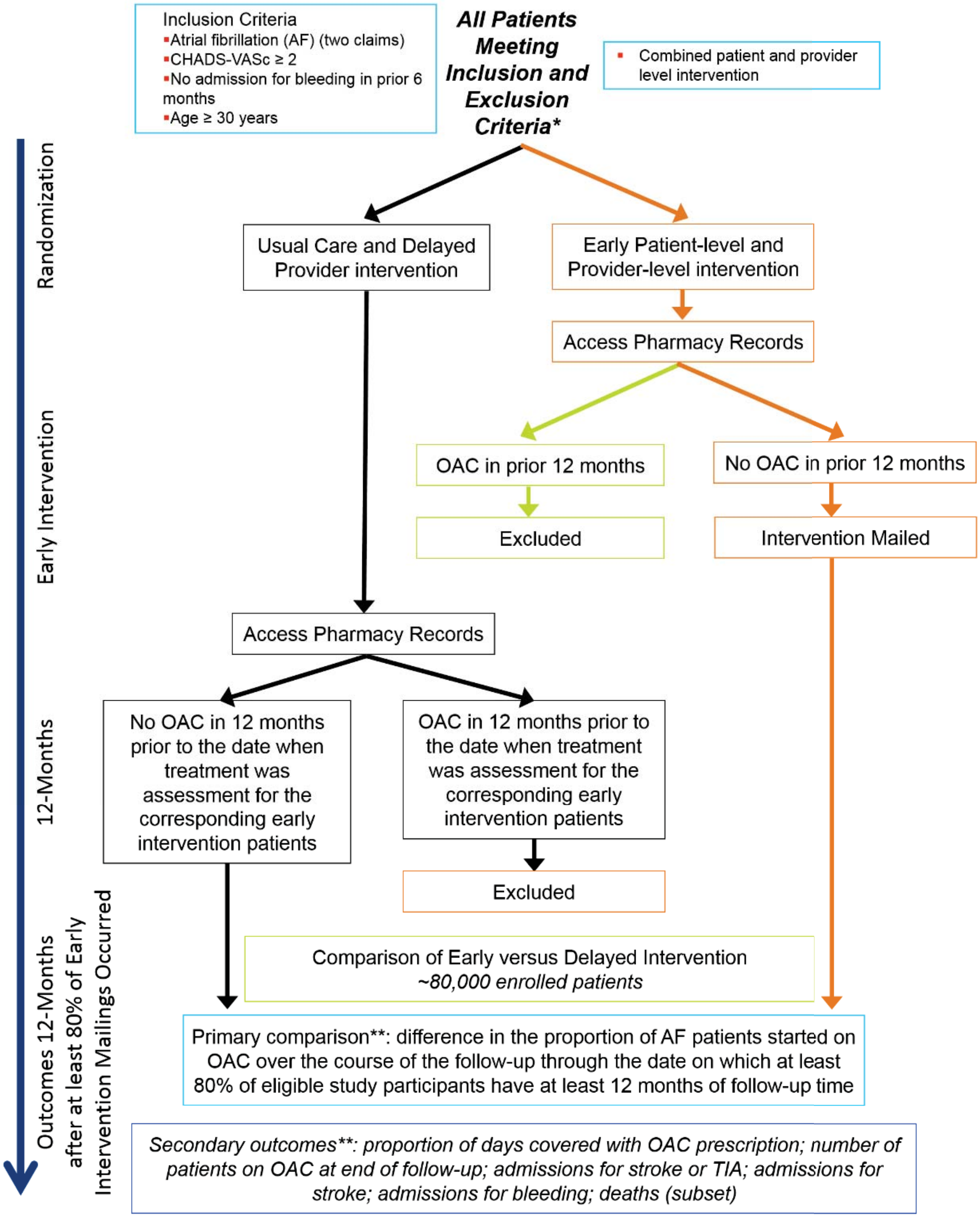

*Baseline characteristics of delayed and early intervention cohorts will be taken from the same time point at randomization from a dataset that is archived at randomization, while exclusion criteria for evidence of OAC medication fill or P2Y12 antagonist use was determined at randomization for the early intervention cohort and approximately 12 months post-randomization for the delayed intervention cohort.
**All possible person-time will be used to assess participants' outcomes (patients will have different duration of follow-up).

For analysis, treatment status is at time of randomization or corresponding early intervention mailing; for the delayed intervention mailing it is prior to mailing.

A schematic diagram below shows the design of the delayed intervention portion of the study:

Figure 2. Design of the delayed intervention portion of the study

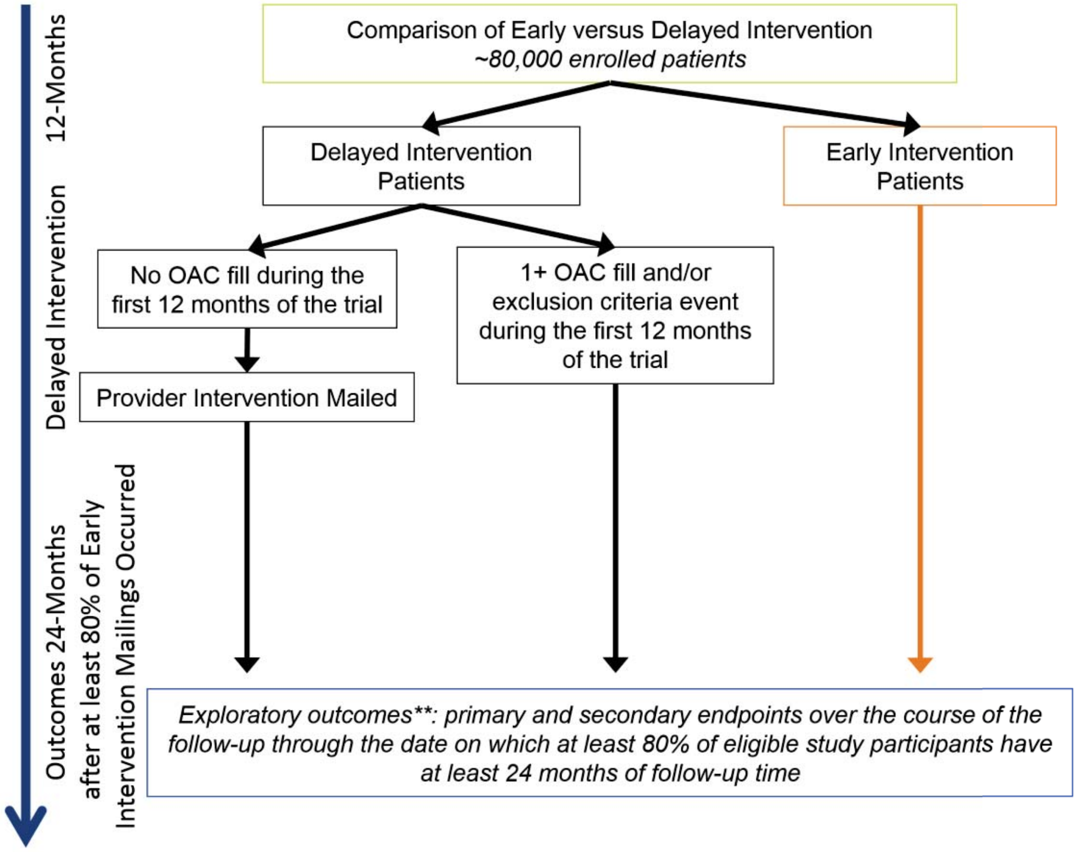

*All possible person-time will be used to assess participants' outcomes (patients will have different duration of follow-up).

For analysis, treatment status is at time of randomization or corresponding early intervention mailing; for the delayed intervention mailing it is prior to mailing.

G. SAMPLE SIZE JUSTIFICATION

1. Primary Endpoint

The following assumptions were used to determine the sample size and power for the primary endpoint assessing the proportion of AF patients with evidence of at least one OAC prescription fill through the date on which at least 80% of eligible study participants have at least 12 months of follow-up time:

1. 33% OAC initiation rate in the delayed intervention arm
2. 38% OAC initiation rate in the early intervention arm (5% absolute improvement in OAC initiation over the 33% OAC initiation expected in the delayed intervention arm over 1-year follow-up)
3. 1-year attrition rate: 30% dropout or lost-to-follow-up
4. Two-sided type I error rate of 0.05
5. Roughly 10,000 patients who meet the inclusion/exclusion criteria will yield more than 99% power to detect a 5% absolute difference

Table 2. Sample size and power for the primary endpoint

Power	Total sample size (2-arm)	Early Intervention	Delayed Intervention
90%	5610	2805	2805
90%	6910	3455	3455
90%	9718	4859	4859

Figure 3. Total number of patients in a 2-arm study

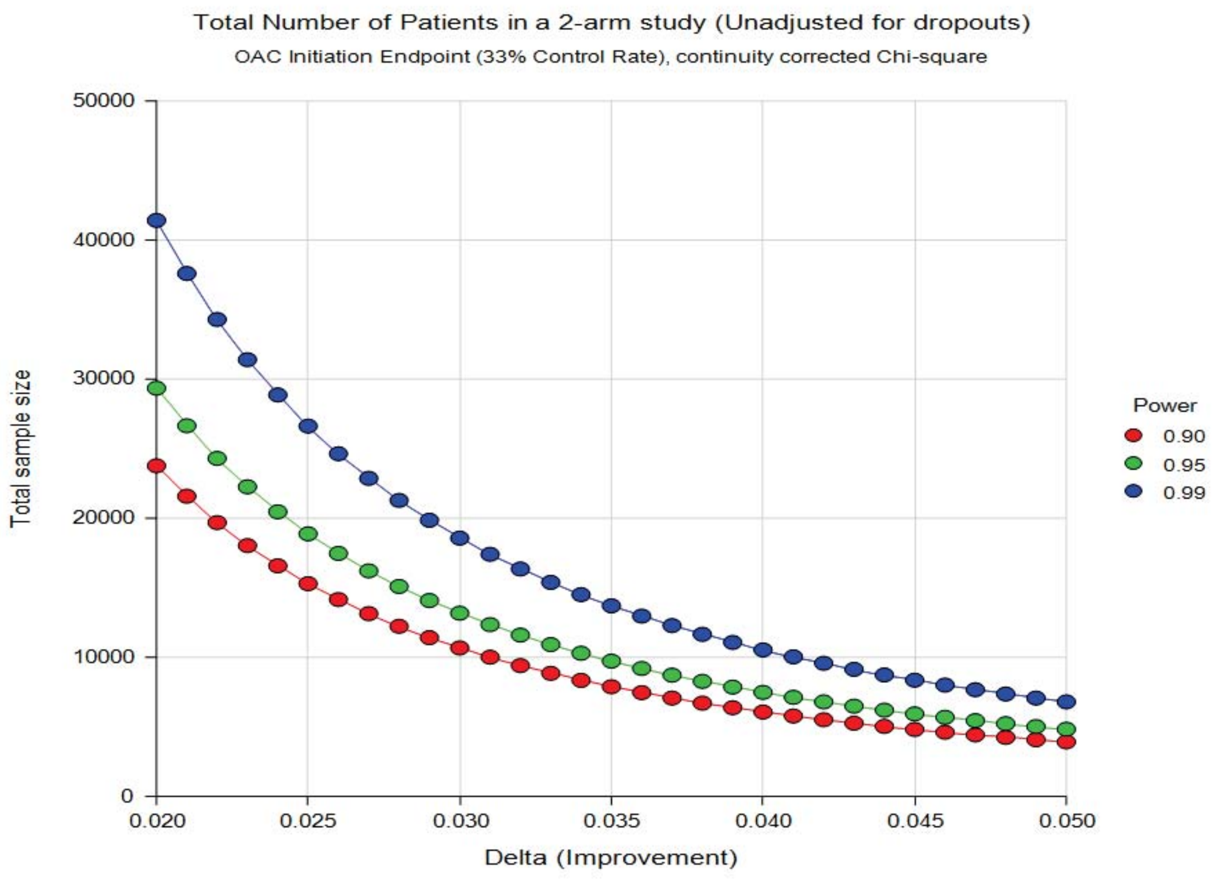

2. Important Secondary Outcome of Stroke or TIA

A study with approximately 80,000 patients could provide reasonable power for stroke or TIA outcome under certain assumptions listed below:

1. 1-year stroke or TIA rate: 18% among patients not treated with OAC
2. 1-year stroke or TIA rate: 7% among patients treated with at least 1 OAC fill
3. Duration of follow-up: 1 year
4. 33% of delayed intervention patients will have at least 1 fill of OAC, meaning the 1 year stroke or TIA rate in the delayed intervention group would be 14.4%
5. 1-year attrition rate: 30% dropout or lost-to-follow-up
6. Two-sided type I error of 0.05
7. If 38% of early intervention patients have at least 1 fill of OAC (meaning the 1 year stroke or TIA rate in the early intervention group would be 13.82%, i.e., an absolute reduction of 0.55%), the study will have 46% power to detect this 0.55% reduction. However, if 40.5% of early intervention patients have at least 1 fill of OAC (meaning the 1 year stroke or TIA rate in the early intervention group would be 13.54%, i.e., an absolute reduction of 0.83%), the study will have 80% power to detect this 0.83% reduction.
8. The sample size has 80% power to detect a 0.5% absolute reduction in stroke, assuming a cumulative 1-year incidence of stroke of 4.2% in control (delayed intervention arm) patients and 3.7% in intervention (early intervention arm) patients. The assumption is that patients not on oral anticoagulation have an annual stroke rate of 5%, and stroke will be reduced by 50% (HR=0.5) in the treated (anticoagulated) population. The 80% power requires that 52% of early intervention arm patients are treated at 1-year as compared to 33% in the control arm.

II. STATISTICAL ANALYSIS

All primary analyses will be based on modified intention-to-treat (mITT) principle (i.e., all identified early intervention patients who met eligibility and were mailed the intervention will be included; using “pseudo” wave assignments, all identified delayed intervention patients who met eligibility at the time of corresponding early mailings will be included). Since the additional exclusions after randomization will be applied in the same way, using the same time points for both the early and delayed intervention groups, we expect there will be no effect from these additional exclusions on the randomization. The mITT analysis will include the following:

- Early intervention members who were mailed a letter, with follow-up beginning on the date of mailing (up to two dates per site and the dates varied by site).
- Delayed intervention members who were not on treatment at the date of mailing (wave assignments were made for the delayed as described earlier in section I.F) with follow-up beginning on that date.

An as-randomized analysis will be performed for the primary endpoint as a sensitivity analysis. Randomization occurred prior to treatment status assessment per the study design. Therefore, the as-randomized analysis will include people who were on treatment as well as others who were not truly eligible for the study (i.e., people who disenrolled, were transitioned to a plan that does not permit their inclusion, had incomplete or invalid addresses, died, or had a “do not contact” status).

Potential sensitivity analysis for mITT analysis: There may be differential loss to follow up between the early and delayed intervention groups because some members did not have valid mailing addresses,

though we did not assess the addresses of the members in the delayed intervention arm at the time of early intervention mailings. It is not possible to retrospectively ascertain the address status of the delayed intervention group as of the date on which mailing would have occurred for that group. We can estimate the magnitude by referring to the early intervention group, and in the early intervention group, we can compare the baseline characteristics and experience of the individuals who had no valid address to those who do.

All possible person-time will be used to assess participants' outcomes. For the time-to-event analysis, patients will be censored from the analysis at the time of death, disenrollment from the health plan, loss of medical or pharmacy coverage, or change in eligibility for inclusion in research based on health plan membership.

Providers may have more than 1 patient in the study – either within the same arm or in both the early and delayed intervention arms. The frequency of this, when provider overlap can be identified, will be reported descriptively and it is expected to be a low proportion of whole population. Depending on the number of patients in this scenario, a sensitivity analysis may be considered to examine the effect.

There were a few variations in the implementation of the early intervention across the Data Partners due to pragmatic issues. The intervention per the protocol is targeted at both member and provider (i.e., the provider who gave most recent AF diagnosis). If the AF provider was a facility in the source data, the educational intervention was applied to the member only, unless the site chose an alternate provider. In the latter case, the intervention was sent to both the member and the alternate provider. The number of patients in each of these variations to the intended intervention will be reported.

A detailed Consort flow diagram will be provided showing the number of patients randomized to the early and delayed intervention groups, the numbers of subjects lost to follow up or excluded from analyses, and the number of subjects evaluable for the key study endpoints (Figure 1). [1]

All analyses will be conducted using SAS version 9.4 or higher software (SAS Institute Inc., Cary, NC). However, version and modules to be used could vary from one Data Partner to another. All tests will be two-sided and a p-value of <0.05 will be considered statistically significant. No multiplicity adjustment will be made.

Figure 4. Consort Flow Diagram

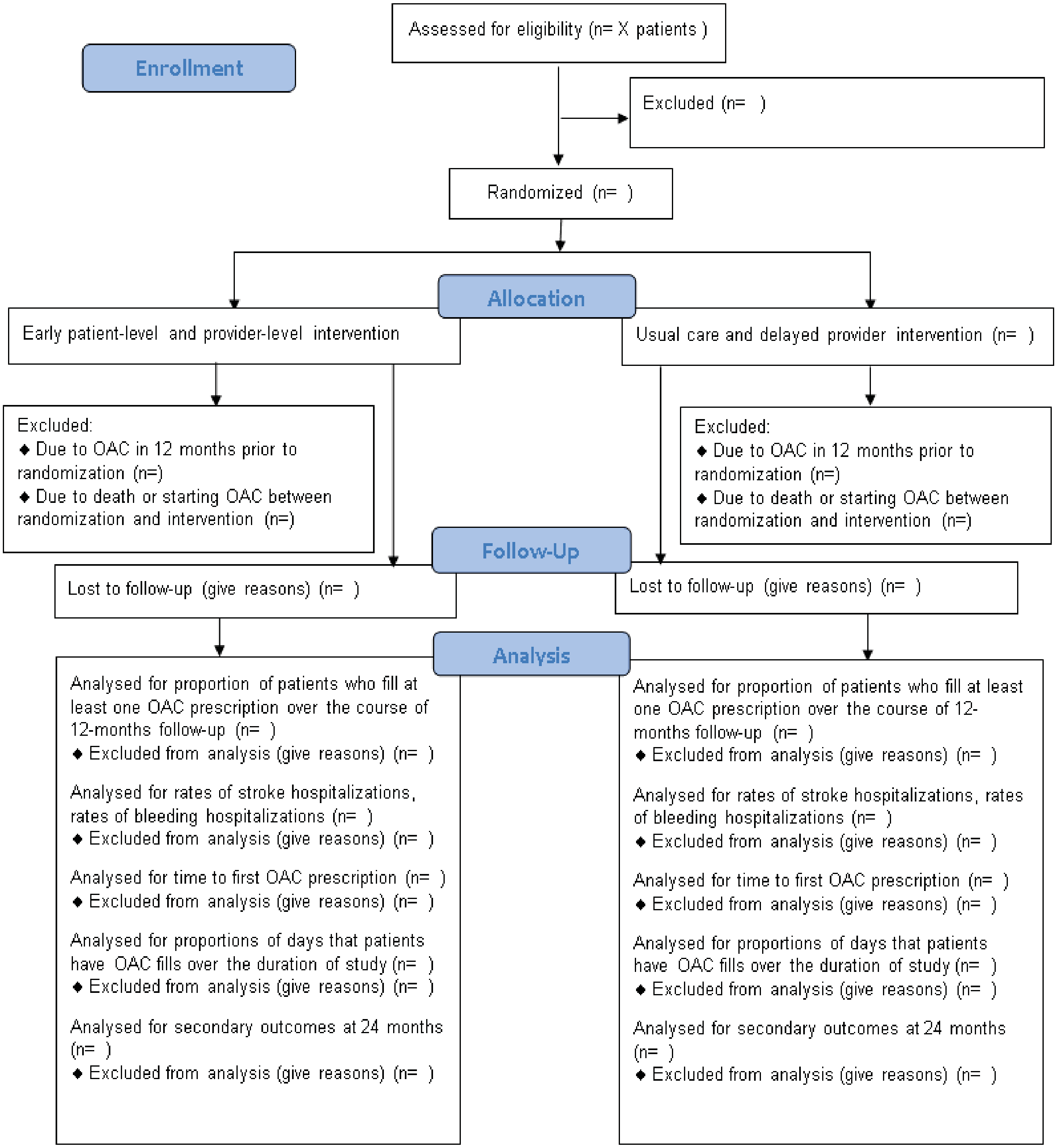

A. METHODS FOR ANALYSIS IN DISTRIBUTED DATA NETWORKS

Patient-level data will be maintained by the Data Partners for all or most analyses. Therefore, analyses specified in this document will be conducted via a distributed SAS programming code developed by the Duke statistical team in collaboration with the study coordinating center at HPHCI as specified in the study table and figure shells. These SAS programs will be shared with HPHCI trial coordinating center for validation, beta testing, and software version compatibility as specified by each Data Partner. Results are expected to be returned by Data Partner to HPHCI and shared with the data coordinating center (DCC) at Duke to conduct an overall statistical analysis across all Data Partners' results. The Duke DCC will have a data use agreement with each Data Partner as necessary in order to receive/access aggregate summary data, which are housed at HPHCI. No Data Partner-specific tables will be shared beyond the coordinating center and Duke DCC, if agreed upon by Data Partners, the analytic team; only data aggregated across sites will be published or made public.

There are several analytic approaches that can be used to perform analysis in a distributed database without requiring patient-level information.[2 3 4 5 6 7 8] Each of these methods requires different types of summary-level information from the participating sites but they generally provide comparable results. We describe these approaches in Appendix D: 1) meta-analysis, 2) case-centered logistic regression, 3) distributed regression. The analysis of primary and secondary outcomes in this study will require using both logistic regression and time-to-event approach. The heterogeneity across Data Partners will need to be assessed. A fixed-effects meta-analysis approach will be the primary statistical method for integrating the findings from each Data Partner. Because case-centered logistic regression and distributed regression methods require a more granular level of data as compared to meta-analysis approach, these methods could be explored as sensitivity analyses depending on the level of data provided by the five Data Partners.

Hereafter, the statistical analysis details will be described based on patient-level data with the understanding that each Data Partner will run these analyses separately and return the results to the HPHCI coordinating center for further analyses.

B. DEMOGRAPHICS AND BASELINE CHARACTERISTICS

Baseline characteristics of the early intervention and delayed intervention cohorts will be based on the claims data at the time of randomization (among those eligible for inclusion in the analysis). Frequency distribution and summary statistics for demographic and baseline variables will be presented by early intervention group, delayed intervention group, and for the overall study population (Table 1 in Appendix A). Key demographic and baseline variables to be summarized include: geographic region, age, sex, risk factors for stroke, risk factors for bleeding, and selected comorbid conditions. Depending on the data availability and effort required by Data Partners, a sensitivity analysis may be conducted to examine the number of patients associated with different provider types (examination of outcomes by provider type may also be considered). Categorical variables will be presented as counts (percentages) and will be compared between groups by using Pearson's chi-square or Fisher's exact test if the count in any cell is less than 5. Continuous variables will be summarized as mean (±SD) and median (25th, 75th percentiles); the comparison between the two groups will be conducted using Wilcoxon rank-sum test for the data within each Data Partner. For the continuous variables in combined study population, only the mean will be summarized. Each Data Partner will run the SAS program(s) distributed by HPHCI, developed with the statistical team at Duke, to generate a summary table for their cohort of patients shown in Tables 1a – 1e in Appendix A (one for each Data Partner) and return the summary table to HPHCI. The same summary table will be generated for entire study population by HPHCI, as shown in Table 1 in Appendix A.

As part of the provider intervention materials, providers had the opportunity to respond and provide an explanation for why their patients were not being treated with OAC. The data collected from these responses will be aggregated by rationale for non-treatment and reported as counts (percentages) by the coordinating center.

C. PRIMARY ENDPOINT DATA ANALYSES

The proportion of patients with evidence of at least one OAC medication fill over the course of the follow-up (through the date on which at least 80% of eligible study participants have at least 12 months of follow-up time) is the primary endpoint. The definition for OAC medication fill will be an OAC medication billing in the outpatient pharmacy claims or at least 4 INR tests or results in the laboratory claims (indicative of OAC use that was not billed through the pharmacy claims data). The primary endpoint will be summarized and compared between the early intervention and delayed intervention arms. Both unadjusted and adjusted (based on available baseline risk factors) analyses will be conducted for the difference in the primary endpoint between the early intervention and delayed intervention arms using the data shown in Table 3. The adjusted analysis will be considered the primary analysis.

1. Adjusted Analysis Model

The logistic regression model will be used for analyzing primary endpoint. Let binary indicator variable T denotes randomized treatment groups, i.e. T=1 indicates early intervention; T=0 indicates delayed intervention. Let π denotes the probability that a patient filling at least at least one OAC medication over the course of the 12-months post intervention. The primary analysis model has the form

$$logit(\pi) = \log\left(\frac{\pi}{1-\pi}\right) = \alpha + \beta_0 \mathrm{T} + \boldsymbol{\beta}'\boldsymbol{\chi}$$

where α is the intercept parameter, $\boldsymbol{\chi}$ is the vector of baseline covariates to be adjusted in the model, listed in Appendix A Table 2. The same set of covariates will be used in the analysis performed by each data partner on their cohort of patients. $\boldsymbol{\beta} = (\beta_1, \dots, \beta_s)'$ is the vector of slope parameters. $\frac{\pi}{1-\pi}$ is the odds of a patient filling at least one OAC medication prescription over the course of the follow-up through the date on which at least 80% of eligible study participants have at least 12 months of follow-up time. This model assumes observations are correlated within each Data Partner "cluster" but not across Data Partners, a "working" correlation structure will be used via generalized estimating equation (GEE).[10]

2. Analysis Results Interpretation

The estimate of β_0 in the model in section C.1 is the logarithm of odds ratio of treatment groups (i.e., the odds of an average patient in the early intervention group filling at least one OAC medication prescription as compared to the odds of an average patient in the delayed intervention group filling at least one OAC prescription over the course of the follow-up through the date on which at least 80% of eligible study participants have at least 12 months of follow-up time). To evaluate whether the early patient-level and provider-level educational interventions increases the proportion of patients with evidence of at least one OAC medication fill over the course of the 12-months post intervention, we will calculate the odds ratio using logistic regression model adjusted for baseline risk factors listed in Appendix A Table 2, with GEE to account for the correlation of responses among the patients from a same service provider. The odds ratio, 95% confidence interval (CI), and p-value will be presented to show whether there is a statistically significant difference in the proportion of patients who fill at least one OAC prescription over the course of the follow-up through the date on which at least 80% of eligible study participants have at least 12 months of follow-up time between the early intervention and

delayed intervention groups. Each Data Partner will run the SAS program(s) distributed by HPHCI, developed with the statistical team at Duke DCC, to perform logistic regression on their cohort of patients and return the parameter estimate (standard error [SE]), estimated of odds ratio, 95% CI, and p-value, shown in Table 3a – 3e in Appendix A (one for each Data Partner), to HPHCI. Meta-analysis methods with inverse-variance weighting on log scale will be used to integrate the results and obtain the estimate of the overall odds ratio, 95% CI and p-value for entire study population, as shown in Table 3 in Appendix A.

There are patients for whom a provider was not identified, and therefore the provider letters were not mailed out. The primary endpoint will be summarized and compared between the patients with provider letter and the patients without provider letter. Each Data Partner will return the descriptive data, shown in Table 4a – 4e in Appendix A to HPHCI. Similar summary table will be generated for entire intervention arm by HPHCI, as shown in Table 4 in Appendix A. To evaluate whether the intervention effect is different between the patients with provider letter and the patients without provider letter, an interaction term between treatment and provider status (an indicator variable of Yes/No for the provider letter received) will be tested in the above multivariable model. Each Data Partner will return the p-value of interaction term to HPHCI. Fisher's method as described below will be used to combine the p-values of test for the interaction term from each Data Partner and generate the p-value for the interaction term for entire cohort.

$$\chi 2_{2k} = -\sum_{i=1}^{k} \ln(p_i)$$

Where p_i is the p-value from *ith* Data Partner.

D. SECONDARY ENDPOINTS DATA ANALYSES

1. OAC Initiation

The time to first OAC initiation, is defined by the first fill date for apixaban, dabigatran, edoxaban, rivaroxaban, or warfarin. If there was no prescription fill for these medications, but the patient had 4 or more INR tests or results documented over the study period, the date of the first INR measurement would be used for initiation of OAC. A Cox proportional hazards[11] model with early intervention vs. delayed intervention as the main effect will be used to model the time to first OAC initiation, after adjusting for baseline risk factors listed in Appendix A Table 2. A robust sandwich covariance estimate or a frailty model will be used to account for the correlation of responses among the patients from a same service provider. The hazard ratio, 95% CI and p-value will be summarized for apixaban, dabigatran, edoxaban, rivaroxaban, or warfarin. Each Data Partner will run the SAS program(s) distributed by HPHCI, developed with the statistical team at Duke DCC, to perform Cox regression model[11] on their cohort of patients and return the estimated hazard ratio, 95% CI and p-value, shown in Table 5a – 5e in Appendix A (one for each Data Partner), to HPHCI. Meta-analysis methods with inverse-variance weighting on log scale will be used to integrate the results and obtain the overall estimate of the hazard ratio, 95% CI and p-value for the entire study population, as shown in Table 5 in Appendix A.

2. OAC Adherence

OAC adherence will be assessed by the proportion of days covered by OAC prescription fills over the duration of the study, or the proportion of days covered. The assumption is that a 30-day or 90-day supply will last for the planned period, even in the case of warfarin, when the length of time that a prescription lasts may be less well defined. Overlapped prescription days will be counted only once, and the days prescribed beyond censor date will be censored accordingly in calculating the total days

covered by OAC. Patients will only be included in this secondary analysis if they had a prescription fill for apixaban, dabigatran, edoxaban, rivaroxaban, or warfarin. Proportion of days covered will be summarized as mean (±SD), and median (25th, 75th percentiles) by early vs. delayed intervention, the comparison between the two groups will be conducted using Wilcoxon rank-sum test, as shown in Table 6 in Appendix A. Each Data Partner will run the SAS program(s) distributed by HPHCI, developed with the statistical team at Duke, to generate a summary table for their cohort of patients shown in Tables 6a – 6e in Appendix A (one for each Data Partner) and return the summary table to HPHCI. For entire study population, the mean of two groups will be generated using meta-analysis methods. Fisher's method as described in Section C will be used to combine the p-values of Wilcoxon rank-sum test from each Data Partner and generate the p-value for entire cohort.

3. Proportion of Patients Actively on OAC at the End of Follow-up

The proportion of patients actively on OAC at the end of follow-up will be summarized and compared between the early intervention and delayed intervention arms. Last Observation Carry Forward will be used if the patient is censored. To evaluate whether the early patient-level and provider-level educational intervention increases the proportion of patients being actively on OAC at the end of follow-up, we will calculate the odds ratio (i.e., the odds of an average patient in the early intervention group being actively on OAC at the end of follow-up as compared to the odds of an average patient in the delayed intervention group being actively on OAC at the end of follow-up) using logistic regression model adjusted for baseline risk factors listed in Appendix A Table 2, with GEE to account for the correlation of responses among the patients from a same service provider. The odds ratio, 95% confidence interval (CI) and p-value will be presented to show whether there is a statistically significant difference in the proportion of patients who are actively on OAC at the end of follow-up between the early intervention and delayed intervention groups. Each Data Partner will run the SAS program(s) distributed by HPHCI, developed with the statistical team at Duke DCC, to perform logistic regression analysis on their cohort of patients and return the parameter estimate (SE), estimated odds ratio, 95% confidence interval (CI) and p-value, shown in Table 7a – 7e in Appendix A (one for each Data Partner), to HPHCI. Meta-analysis methods with inverse-variance weighting on log scale will be used to integrate the results and obtain the estimate of the overall odds ratio, 95% confidence interval and p-value for entire study population, as shown in Table 7 in Appendix A.

4. Clinical Outcomes

Claims data (ICD-10-CM codes used for defining these outcomes are listed in Appendix C) will be used to define the following clinical outcomes:

- Hospitalization for ischemic stroke or unknown stroke
- Hospitalization for hemorrhagic stroke
- Hospitalization for any bleeding
- Composite of ischemic or hemorrhagic stroke
- Composite of ischemic stroke, hemorrhagic stroke, and hospitalization for any bleeding
- All-cause in-hospital death

For each of these outcomes, time-to-event methodology will be implemented. Kaplan-Meier estimator[12] will be used to estimate the probability of occurrence at 12 months of follow-up and the log-rank test[13] will be used to compare the survival curves. The Cox proportional hazards model with early intervention vs. delayed intervention as main effect will be used to model the time to event, after adjusting for baseline risk factors listed in Appendix A Table 2. The hazard ratio, 95% CI and p-value will be presented to summarize the difference in the risk of clinical outcome between early intervention and delayed intervention groups. In-hospital death or medically attended death will be collected through claims data.

Depending on number of patients and events, the comparison of the stroke rate between early and delayed intervention may be examined separately in patients who have 1 OAC fill and multiple OAC fills. This will be descriptive only and will not include formal statistical testing.

Each Data Partner will run SAS program(s) distributed by HPHCI, developed with the statistical team at Duke DCC, to perform analysis on their cohort of patients and return the results to HPHCI. For the comparison of probability of occurrence, each Data Partner will return the estimate of probability of occurrence, standard error, 95% CI by early intervention vs. delayed intervention, and p-value, as shown in Table 8a - 8e in Appendix A (one for each Data Partner). For the comparison of risk, each Data Partner will return the parameter estimate (SE), hazard ratio, 95% CI and p-value, as shown in in Appendix A Table 9a - 9e (one for each Data Partner). The statistical team will use meta-analysis methods to integrate the results and obtain the results for entire study population, as shown in Tables 8 and 9 in Appendix A.

5. Health Care Utilization

The total counts of health care utilization (number of outpatient visits, emergency department visits, hospital admissions, and days hospitalized) at the end of follow-up time, including AF and non-AF related care, will be summarized by early intervention and delayed intervention group, as shown in Table 10 in Appendix A.

III. REFERENCES

1. Zwarenstein M, Treweek S, Gagnier JJ, Altman DG, Tunis S, Haynes B, Oxman AD, Moher D for the CONSORT and Pragmatic Trials in Healthcare (Practihc) group. Improving the reporting of pragmatic trials: an extension of the CONSORT statement. BMJ 2008; 337; a2390.

2. Toh S, Reichman ME, Houstoun M, Ding X, Fireman BH, Gravel E, Levenson M, Li L, Moyneur E, 21` 43 Shoaibi A, Zornberg G, Hennessy S. Multivariable confounding adjustment in distributed data networks without sharing of patient-level data. Pharmacoepidemiology and Drug Safety. 2013 Nov; 22(11):1171-7.

3. Toh S, Gagne JJ, Rassen JA, Fireman BH, Kulldorff M, Brown JS. Confounding adjustment in comparative effectiveness research conducted within distributed research networks.

Med Care. 2013 Aug; 51(8 Suppl 3): S4-10.

4. Toh S, Shetterly S, Powers JD, Arterburn D. Privacy-preserving analytic methods for multisite comparative effectiveness and patient-centered outcomes research. Med Care. 2014 Jul; 52 (7):664-8.

5. DerSimonian R, Lai N. Meta-analysis in clinical trials. *Control Clin Trials*. 1986; 7: 177–188.

6. Fireman B, Lee J, Lewis N, Bembom O, van der Laan M, Baxter R.. Influenza vaccination and mortality: differentiating vaccine effects from bias. Am J Epidemiol. 2009 Sep 1;170(5):650-6.

7. Karr AF, Lin X, Sanil AP, Reiter JP. Secure regression on distributed databases. *J Comput Graph Stat*

2005;**14**(2):263-279.

8. Fienberg SE, Fulp WJ, Slavković AB, Wrobel TA. "Secure" log-linear and logistic regression analysis of

distributed databases. *Lect Notes Comput Sci* 2006;**2006**(4302):277-290.

9. Her QL, Malenfant JM, Malek S, Vilk Y, Young J, Li L, Brown J, Toh S. A query workflow design to perform automatable distributed regression analysis in large distributed data networks. *eGEMs: The Journal of Electronic Health Data and Methods* (in press)

10. Liang, K. Y. and Zeger, S. L. (1986) longitudinal data analysis using generalized linear models. Biometrika, 73, 13-22.

11. Cox DR. Regression models and life-tables. J Royal Statist Soc B 1972; 34:187-220.

12. Kaplan EL, Meier P. Nonparametric estimation from incomplete observations. J Am Statist Assn 1958;53:457-481.

13. Kalbfleisch JD, Prentice RL. The Statistical Analysis of Failure Time Data. 2nd edition. 2002, John Wiley & Sons, Inc. Hoboken, New Jersey.

IV. APPENDIX

A. TABLES

For all tables below, the study coordinating center at HPHCI will receive Data Partner-specific tables. The coordinating center will generate aggregated tables across Data Partners (shown below), using meta-analysis methods where appropriate (for continuous variables in aggregated tables, only mean will be generated); these will be shared with the Duke DCC statistical team. Duke DCC will have a data-use agreement with each Data Partner as necessary in order to receive/access aggregate summary data, which are housed at HPHCI. No Data Partner-specific tables will be shared beyond the coordinating center and Duke DCC, if agreed upon by Data Partners, the analytic team; only data aggregated across sites will be published or made public.

Appendix A Table 1. Baseline characteristics of all patients by early intervention vs. delayed intervention

	Early intervention (N=XXXX)	Delayed intervention (N=XXXX)	Overall (N=XXXX)
**Demographics**			
Age			
N			
Mean (SD)			
Median (Min, 25th, 75th, Max)			
30-34 yr, %			
35-39, %			
40-44, %			
45-49, %			
50-54, %			
55-59, %			
60-64, %			
65-69, %			
70-74, %			
75-79, %			
≥ 80, %			
Sex			
N			
Female, %			
Region			
New England, %			
Mid-Atlantic, %			
South-Atlantic, %			
Midwest, %			
Mountain, %			
Pacific, %			
Unknown, %			
**Medical history**			
History of anemia?			

	Early intervention (N=XXXX)	Delayed intervention (N=XXXX)	Overall (N=XXXX)
N			
Yes, %			
History of hypertension?			
N			
Yes, %			
History of diabetes?			
N			
Yes, %			
History of hospitalization for any bleeding?			
N			
Yes, %			
History of peripheral vascular disease?			
N			
Yes, %			
History of prior cerebrovascular disease?			
N			
Yes, %			
History of heart failure?			
N			
Yes, %			
History of kidney disease?			
N			
Yes, %			
History of MI			
N			
Yes, %			
History of CABG			
N			
Yes, %			
CHA_2DS_2 VASc score			
N			
Mean (SD)			
Median (25th, 75th)			
0, %			
1, %			
2, %			
3, %			
4, %			
5, %			
6, %			
7, %			
8, %			
9, %			

	Early intervention (N=XXXX)	Delayed intervention (N=XXXX)	Overall (N=XXXX)
Bleeding risk score: ATRIA score			
Low (≤3), %			
Intermediate (4), %			
High (≥5), %			
Hospitalization in the prior 6 months			
0, %			
1, %			
2, %			
≥3, %			

For Tables 3 through 10: The SAP workplan(s) run at each Data Partner sites will generate Partner-specific tables of events (per outcome, for early vs delayed intervention) and unadjusted and adjusted model results. These will be returned to the coordinating center; results will be aggregated and meta-analyses will be run on site-specific models to generate final aggregated results.

Appendix A Table 2. Baseline covariates to be adjusted in the models described in Sections II.C, II.D.1, II.D.2, II.D.3, and II.D.4

**Demographics**
Age (continuous)
Sex (male vs. female)
Region (New England vs. Mid-Atlantic vs. South-Atlantic vs. Midwest vs. Mountain vs. Pacific)
**Medical history**
History of anemia (yes vs. no)
History of hypertension (yes vs. no)
History of diabetes (yes vs. no)
History of hospitalization for any bleeding (yes vs. no)
History of peripheral vascular disease (yes vs. no)
History of prior cerebrovascular disease (yes vs. no)
History of heart failure (yes vs. no)
History of kidney disease (yes vs. no)
History of MI
History of CABG
Bleeding risk score: ATRIA score (Low (≤3) vs. Intermediate (4) vs. High (≥5))
Hospitalization in the prior 6 months (0 vs. 1 vs. 2 vs. ≥3)

Appendix A Table 3. Odds ratio of filling at least one OAC medication prescription over the course of the 12-months of follow-up: early intervention vs. delayed intervention (replicated at each Data Partner)

	Early Intervention, n/N (%)	Delayed Intervention, n/N (%)	Parameter Estimate (SE)	Odds Ratio (95% CI)	p-value
OAC Prescription at 1-year					

Appendix A Table 4. Numbers and proportions of filling at least one OAC medication prescription over the course of the 12-months of follow-up in intervention arm: patients with provider letter vs. patients without provider letter (replicated at each Data Partner)

	Patients with Provider Letter, n/N (%)	Patients without Provider Letter, n/N (%)	p-value
OAC Prescription at 1-year			

Appendix A Table 5. Hazard ratio of OAC initiation: early intervention vs. delayed intervention (replicated at each Data Partner)

	Early Intervention, n/N (%)	Delayed Intervention, n/N (%)	Parameter Estimate (SE)	Hazard Ratio (95% CI)	p-value
OAC initiation					

Appendix A Table 6. Proportion of days covered: early intervention vs. delayed intervention (replicated at each Data Partner)

	Early Intervention	Delayed Intervention	p-value
N			
Mean (SD)			
Median (Min, 25th, 75th, Max)			

Appendix A Table 7. Proportion of patients actively on OAC at the end of follow-up: early intervention vs. delayed intervention (replicated at each Data Partner)

	Early Intervention, n/N (%)	Delayed Intervention, n/N (%)	Parameter Estimate (SE)	Odds Ratio (95% CI)	p-value
Actively on OAC at the end of follow-up					

Appendix A Table 8. Probability of occurrence at 1-year: early intervention vs. delayed intervention (replicated at each Data Partner)

Outcomes	Early Intervention, n/N (%)	Delayed Intervention, n/N (%)	Early Intervention, Probability (SE) (95% CI)	Delayed Intervention, Probability (SE) (95% CI)	p-value
Hospitalization for ischemic stroke or unknown stroke					
Hospitalization for hemorrhagic stroke					
Hospitalization for any bleeding					
Composite of ischemic or hemorrhagic stroke					
Composite of ischemic stroke, hemorrhagic stroke, and hospitalization for any bleeding					
All-cause in-hospital death					

Appendix A Table 9. Hazard ratio of each outcome: early intervention vs. delayed intervention (replicated at each Data Partner)

Outcomes	Early Intervention, n/N (%)	Delayed Intervention, n/N (%)	Parameter Estimate (SE)	Hazard Ratio (95% CI)	p-value
Hospitalization for ischemic stroke or unknown stroke					
Hospitalization for hemorrhagic stroke					
Hospitalization for any bleeding					
Composite of ischemic or hemorrhagic stroke					
Composite of ischemic stroke, hemorrhagic stroke, and hospitalization for any bleeding					
All-cause in-hospital death					

Appendix A Table 10. Health care utilization at 12 months: early Intervention vs. delayed Intervention (replicated at each Data Partner)

	Early Intervention	Delayed Intervention	p-value
Visit Type			
N (Total visits)			
Patients with outpatient visits, % (total outpatient visits)			
Patients with emergency department visits, % (total ED visits)			
Patients with hospital admissions, % (total hospitalizations)			
Days hospitalized			
N (Total)			
Mean (SD)			
Median (25th, 75th)			

B. CONSORT 2010 WORKSHEET FOR RANDOMIZED TRIALS

CONSORT 2010 checklist of information to include when reporting a randomized trial

Section/Topic	Item No	Checklist item	Reported on page No
**Title and abstract**			
	1a	Identification as a randomized trial in the title	
	1b	Structured summary of trial design, methods, results, and conclusions (for specific guidance see CONSORT for abstracts)	
**Introduction**			
Background and objectives	2a	Scientific background and explanation of rationale	
	2b	Specific objectives or hypotheses	
**Methods**			
Trial design	3a	Description of trial design (such as parallel, factorial) including allocation ratio	
	3b	Important changes to methods after trial commencement (such as eligibility criteria), with reasons	
Participants	4a	Eligibility criteria for participants	
	4b	Settings and locations where the data were collected	
Interventions	5	The interventions for each group with sufficient details to allow replication, including how and when they were actually administered	
Outcomes	6a	Completely defined pre-specified primary and secondary outcome measures, including how and when they were assessed	
	6b	Any changes to trial outcomes after the trial commenced, with reasons	
Sample size	7a	How sample size was determined	
	7b	When applicable, explanation of any interim analyses and stopping guidelines	
Randomisation:			
Sequence generation	8a	Method used to generate the random allocation sequence	
	8b	Type of randomization; details of any restriction (such as blocking and block size)	
Allocation concealment mechanism	9	Mechanism used to implement the random allocation sequence (such as sequentially numbered containers), describing any steps taken to conceal the sequence until interventions were assigned	
Implementation	10	Who generated the random allocation sequence, who enrolled participants, and who assigned participants to interventions	
Blinding	11a	If done, who was blinded after assignment to interventions (for example, participants, care providers, those assessing outcomes) and how	
	11b	If relevant, description of the similarity of interventions	

Section/Topic	Item No	Checklist item	Reported on page No
Statistical methods	12a	Statistical methods used to compare groups for primary and secondary outcomes	
	12b	Methods for additional analyses, such as subgroup analyses and adjusted analyses	
**Results**			
Participant flow (a diagram is strongly recommended)	13a	For each group, the numbers of participants who were randomly assigned, received intended treatment, and were analyzed for the primary outcome	
	13b	For each group, losses and exclusions after randomization, together with reasons	
Recruitment	14a	Dates defining the periods of recruitment and follow-up	
	14b	Why the trial ended or was stopped	
Baseline data	15	A table showing baseline demographic and clinical characteristics for each group	
Numbers analysed	16	For each group, number of participants (denominator) included in each analysis and whether the analysis was by original assigned groups	
Outcomes and estimation	17a	For each primary and secondary outcome, results for each group, and the estimated effect size and its precision (such as 95% confidence interval)	
	17b	For binary outcomes, presentation of both absolute and relative effect sizes is recommended	
Ancillary analyses	18	Results of any other analyses performed, including subgroup analyses and adjusted analyses, distinguishing pre-specified from exploratory	
Harms	19	All important harms or unintended effects in each group (for specific guidance see CONSORT for harms)	
**Discussion**			
Limitations	20	Trial limitations, addressing sources of potential bias, imprecision, and, if relevant, multiplicity of analyses	
Generalizability	21	Generalizability (external validity, applicability) of the trial findings	
Interpretation	22	Interpretation consistent with results, balancing benefits and harms, and considering other relevant evidence	
**Other information**			
Registration	23	Registration number and name of trial registry	
Protocol	24	Where the full trial protocol can be accessed, if available	
Funding	25	Sources of funding and other support (such as supply of drugs), role of funders	

C. ICD-10 CODES FOR DEFINING CLINICAL OUTCOMES

The below ICD-10-CM codes will be used to define clinical outcomes during analyses (list is not final).

a. **Ischemic stroke or unknown stroke:** diagnosis of
    1. I63.x Cerebral infarction, including
        i. I63.0 Cerebral infarction due to thrombosis of precerebral arteries
        ii. I63.1 Cerebral infarction due to embolism of precerebral arteries
        iii. I63.2 Cerebral infarction due to unspecified occlusion or stenosis of precerebral arteries
        iv. I63.3 Cerebral infarction due to thrombosis of cerebral arteries
        v. I63.4 Cerebral infarction due to embolism of cerebral arteries
        vi. I63.5 Cerebral infarction due to unspecified occlusion or stenosis of cerebral arteries
        vii. I63.6 Cerebral infarction due to cerebral venous thrombosis, nonpyogenic
        viii. I63.8 Other cerebral infarction
        ix. I63.9 Cerebral infarction, unspecified
    2. I67.81 Acute cerebrovascular insufficiency
    3. I67.82 Cerebral ischemia
    4. I67.89 Other cerebrovascular disease
    5. I67.9 Cerebrovascular disease, unspecified
    6. G45.x Transient cerebral ischemic attacks and related syndromes, including
        i. G45.0 Vertebro-basilar artery syndrome
        ii. G45.1 Carotid artery syndrome (hemispheric)
        iii. G45.2 Multiple and bilateral precerebral artery syndromes
        iv. G45.3 Amaurosis fugax
        v. G45.4 Transient global amnesia
        vi. G45.8 Other transient cerebral ischemic attacks and related syndromes
        vii. G45.9 Transient cerebral ischemic attack, unspecified
    7. without primary diagnosis of Intracranial injury (S06.x)

b. **Hemorrhagic stroke:** diagnosis of
    1. I60.x Nontraumatic subarachnoid hemorrhage
    2. I61.x Nontraumatic intracerebral hemorrhage
    3. I62.x Other and unspecified nontraumatic intracranial hemorrhage
    4. without primary diagnosis of Intracranial injury (S06.x)

c. **Hospitalization for any bleeding:** see ICD-10-CM codes in "any hemorrhage" category in cohort identification code list (of which GI bleeding is a subset)

d. **Hospitalization for GI bleeding:**
    1. I85.01 Esophageal varices with bleeding
    2. I85.11 Secondary esophageal varices with bleeding
    3. K22.11 Ulcer of esophagus with bleeding
    4. K22.6 Gastro-esophageal laceration-hemorrhage syndrome
    5. Gastric ulcer
        i. K25.0 Acute with hemorrhage
        ii. K25.2 Acute with hemorrhage and perforation
        iii. K25.4 Chronic or unspecified with hemorrhage
        iv. K25.6 Chronic or unspecified with hemorrhage and perforation
    6. Duodenal ulcer
        i. K26.0 Acute with hemorrhage

ii. K26.2 Acute with hemorrhage and perforation
iii. K26.4 Chronic or unspecified with hemorrhage
iv. K26.6 Chronic or unspecified with hemorrhage and perforation

7. Peptic ulcer, site unspecified
    i. K27.0 Acute with hemorrhage
    ii. K27.2 Acute with hemorrhage and perforation
    iii. K27.4 Chronic or unspecified with hemorrhage
    iv. K27.6 Chronic or unspecified with hemorrhage and perforation
8. Gastrojejunal ulcer
    i. K28.0 Acute with hemorrhage
    ii. K28.2 Acute with hemorrhage and perforation
    iii. K28.4 Chronic or unspecified with hemorrhage
    iv. K28.6 Chronic or unspecified with hemorrhage and perforation
    v. K29.01 Acute gastritis with bleeding
    vi. K29.21 Alcoholic gastritis with bleeding
    vii. K29.31 Chronic superficial gastritis with bleeding
    viii. K29.41 Chronic atrophic gastritis with bleeding
    ix. K29.51 Unspecified chronic gastritis with bleeding
    x. K29.61 Other gastritis with bleeding
    xi. K29.71 Gastritis, unspecified with bleeding
    xii. K29.81 Duodenitis, with bleeding
    xiii. K29.91 Gastroduodenitis, unspecified, with bleeding
9. K31.811 Angiodysplasia of stomach and duodenum with bleeding
10. K31.82 Dieulafoy lesion (hemorrhagic) of stomach and duodenum
11. K57.01 Diverticulitis of small intestine with perforation and abscess with bleeding
12. K57.11 Diverticulosis of small intestine without perforation or abscess with bleeding
13. K57.13 Diverticulitis of small intestine without perforation or abscess with bleeding
14. K57.21 Diverticulitis of large intestine with perforation and abscess with bleeding
15. K57.31 Diverticulosis of large intestine without perforation or abscess with bleeding
16. K57.33 Diverticulitis of large intestine without perforation or abscess with bleeding
17. K57.41 Diverticulitis of both small and large intestine with perforation and abscess with bleeding
18. K57.51 Diverticulosis of both small and large intestine without perforation or abscess with bleeding
19. K57.53 Diverticulitis of both small and large intestine without perforation or abscess with bleeding
20. K57.81 Diverticulitis of intestine, part unspecified, with perforation and abscess with bleeding
21. K57.91 Diverticulosis of intestine, part unspecified, without perforation or abscess with bleeding
22. K57.93 Diverticulitis of intestine, part unspecified, without perforation or abscess with bleeding
23. K62.5 Hemorrhage of anus and rectum
24. K63.81 Dieulafoy lesion of intestine
25. K92.0 Hematemesis
26. K92.1 Melena
27. K92.2 Gastrointestinal hemorrhage, unspecified

D. ANALYSIS METHODS FOR DISTRIBUTED NETWORKS

1. Meta-analysis

In meta-analysis, each Data Partner estimates the effect and their variance (or other information needed to calculate the weight) using pre-specified models on their own individual-level data and send these to the coordinating center. Then, the overall estimated effect and 95% confidence interval is derived by pooling site-specific estimates. A commonly used weight is inverse of variance on the log scale of the estimate. Meta-analysis method requires the least amount of data sharing and is flexible with respect the types of study design and analysis. However, it is the least flexible with respect to the subgroup and sensitivity analyses and requires greatest degree of programing/analysis ability at each participating site.

2. Case-centered Logistic Regression

In this method, each Data Partner transfers an aggregated dataset to the coordinating center that includes 1 record per risk set. Each risk set is anchored by a case (patient with the outcome of interest) and comprised of the cases and comparable individuals at risk of the outcome at the time the case occurs. Each record includes a binary variable indicating whether the patient diagnosed with the outcome is exposed to the treatment and the log odds of the site-specific proportion of exposed patients in the risk set. Confounding adjustment will be conducted through stratification. Specifically, if the number of imbalanced covariates is small, we will create strata that are defined by these covariates within each site; the risk set for a given case will be at-risk individuals who are within the same covariate stratum as the case. If the number of imbalanced covariates is large, we will create propensity score (PS) strata. The PS will be estimated within each site, and the risk set for a given case will be at-risk individuals who are within the same propensity score stratum as the case. The statistical team fits a logistic regression model with indicator variable as the dependent variable and log odds as the independent variable. It is shown that this method maximized the same likelihood as a stratified Cox model using patient–level data. Thus, it is appropriate for study designs that needs to be analyzed using Cox proportional hazards model.

3. Distributed Regression

Distributed regression is a suite of methods that enable researchers to conduct multi-database multivariable-adjusted regression analysis without the need to centrally combine all individual-level data from participating sites. It performs the same numeric algorithm as standard ordinary least squares regression but uses only summary statistics for computation. By following the same computation process, distributed regression and pooled individual-level data analysis produce statistically equivalent results. With distributed regression, we can adjust for imbalanced covariates directly in the outcome regression models.
