# Supplementary material for: Effect of Mailing Educational Material to Patients With Atrial Fibrillation and Their Clinicians on Use of Oral Anticoagulants: A Randomized Clinical Trial
Source: JAMA Netw Open. 2022 May 31;5(5):e2214321. doi: 10.1001/jamanetworkopen.2022.14321 (PMC9157265; doi:10.1001/jamanetworkopen.2022.14321)
Supplement: Supplement 2. — eTable 1. Baseline Characteristics of the Sensitivity Analysis Population eTable 2. Health Care Resource Utilization for the Modified Intention to Treat Population eFigure 1. Patient Educational Intervention Mailing Materials eFigure 2. Provider Educational Intervention Mailing Materials eFigure 3. Sensitivity Analysis of Primary Endpoint Results eFigure 4. Sensitivity Analysis of Primary Endpoint Subgroups eFigure 5. Sensitivity Analysis of Secondary Clinical Outcomes [file jamanetwopen-e2214321-s002.pdf]

Supplementary Online Content

Pokorney SD, Cocoros N, Al-Khalidi HR, et al. Effect of mailing educational material to patients with atrial fibrillation and their clinicians on use of oral anticoagulants: a randomized clinical trial. *JAMA Netw Open.* 2022;5(5):e2214321. doi:10.1001/jamanetworkopen.2022.14321

eTable 1. Baseline Characteristics of the Sensitivity Analysis Population
eTable 2. Health Care Resource Utilization for the Modified Intention to Treat Population
eFigure 1. Patient Educational Intervention Mailing Materials
eFigure 2. Provider Educational Intervention Mailing Materials
eFigure 3. Sensitivity Analysis of Primary Endpoint Results
eFigure 4. Sensitivity Analysis of Primary Endpoint Subgroups
eFigure 5. Sensitivity Analysis of Secondary Clinical Outcomes

This supplementary material has been provided by the authors to give readers additional information about their work.

© 2022 Pokorney SD et al. *JAMA Network Open.*

eTable 1. Baseline Characteristics of the Sensitivity Analysis Population

	Early Intervention (N=28812)	Delayed Intervention (N=31293)
Age*	78.50 (9.57)	78.79 (9.54)
Age < 30 yr	1 (0.00%)	0 (0.00%)
Age 30-34 yr	8 (0.03%)	5 (0.02%)
Age 35-39 yr	20 (0.07%)	25 (0.08%)
Age 40-44 yr	48 (0.17%)	53 (0.17%)
Age 45-49 yr	123 (0.43%)	129 (0.41%)
Age 50-54 yr	299 (1.04%)	260 (0.83%)
Age 55-59 yr	608 (2.11%)	609 (1.95%)
Age 60-64 yr	1003 (3.48%)	1093 (3.49%)
Age 65-69 yr	2607 (9.05%)	2738 (8.75%)
Age 70-74 yr	5318 (18.46%)	5682 (18.16%)
Age 75-79 yr	5977 (20.74%)	6605 (21.11%)
Age 80-84 yr	5348 (18.56%)	5621 (17.96%)
Age 85-89 yr	4172 (14.48%)	4678 (14.95%)
Age 90 and older	3280 (11.38%)	3795 (12.13%)
Male	14781 (51.30%)	16243 (51.91%)
Region: New England	713 (2.47%)	892 (2.85%)
Region: Mid-Atlantic	1226 (4.26%)	1378 (4.40%)
Region: South-Atlantic	16008 (55.56%)	16674 (53.28%)
Region: Midwest	7027 (24.39%)	8386 (26.80%)
Region: Mountain	1536 (5.33%)	1575 (5.03%)
Region: Pacific	2016 (7.00%)	2126 (6.79%)
Region: Unknown/missing	286 (0.99%)	262 (0.84%)
History of anemia	15636 (54.27%)	17074 (54.56%)
History of hypertension	27370 (95.00%)	29776 (95.15%)
History of diabetes	11470 (39.81%)	12302 (39.31%)
History of hospitalization for any bleeding (ever)	5746 (19.94%)	6449 (20.61%)
History of any bleeding hospitalization in prior 183 days	446 (1.55%)	526 (1.68%)
History of GI bleeding hospitalization (ever)	2879 (9.99%)	3247 (10.38%)
History of GI bleeding hospitalization in prior 183 days	222 (0.77%)	257 (0.82%)
History of peripheral vascular disease	7216 (25.05%)	7870 (25.15%)
History of prior cerebrovascular disease	6479 (22.49%)	7065 (22.58%)
History of heart failure	11738 (40.74%)	12790 (40.87%)
History of kidney disease	2295 (7.97%)	2436 (7.78%)
Dialysis	802 (2.78%)	858 (2.74%)
History of MI	3629 (12.60%)	3968 (12.68%)
History of CABG	4237 (14.71%)	4717 (15.07%)
History of coronary stent	1710 (5.94%)	1915 (6.12%)
CHA2DS2 VASc score*	4.61 (1.69)	4.62 (1.67)
CHA2DS2 VASc score = 1	16 (0.06%)	25 (0.08%)
CHA2DS2 VASc score = 2	2896 (10.05%)	2965 (9.47%)
CHA2DS2 VASc score = 3	5288 (18.35%)	5687 (18.17%)

© 2022 Pokorney SD et al. *JAMA Network Open.*

	**Early Intervention (N=28812)**	**Delayed Intervention (N=31293)**
CHA2DS2 VASc score = 4	6634 (23.03%)	7266 (23.22%)
CHA2DS2 VASc score = 5	5781 (20.06%)	6397 (20.44%)
CHA2DS2 VASc score = 6	4019 (13.95%)	4436 (14.18%)
CHA2DS2 VASc score = 7	2380 (8.26%)	2633 (8.41%)
CHA2DS2 VASc score = 8	1385 (4.81%)	1470 (4.70%)
CHA2DS2 VASc score = 9	413 (1.43%)	414 (1.32%)
Bleeding risk score: ATRIA score <= 3	10846 (37.64%)	11666 (37.28%)
Bleeding risk score: ATRIA score = 4	3956 (13.73%)	4258 (13.61%)
Bleeding risk score: ATRIA score >=5	14010 (48.63%)	15369 (49.11%)
Number of Hospitalizations in the prior 6 months: 0	26022 (90.32%)	28109 (89.83%)
Number of Hospitalizations in the prior 6 months: 1	2148 (7.46%)	2438 (7.79%)
Number of Hospitalizations in the prior 6 months: 2	447 (1.55%)	535 (1.71%)
Number of Hospitalizations in the prior 6 months: >=3	195 (0.68%)	211 (0.67%)
Follow-up Time*	433.65 (137.56)	435.52 (140.04)

© 2022 Pokorney SD et al. *JAMA Network Open.*

eTable 2. Health Care Resource Utilization for the Modified Intention to Treat Population

	Intervention (N=23,546)	Control (N=23,787)	P Value
Total # of encounters per arm	951217	963631	.
# patients with ≥1 encounter	22,543 (95.74%)	22,705 (95.45%)	0.093
Total # of encounters at patient level*	40.40 (39.45)	40.51 (39.51)	0.587
# patients with ≥1 outpatient/ambulatory encounter	22,525 (95.66%)	22,694 (95.41%)	0.137
# of outpatient/ambulatory encounters*	38.17 (36.74)	38.27 (36.81)	0.613
Days from mITT start date to first outpatient/ambulatory encounter*	32.36 (47.99)	32.76 (49.42)	0.279
# patients with ≥1 ED encounter	7,942 (33.73%)	8,082 (33.98%)	0.993
# of ED encounters*	0.66 (1.50)	0.69 (1.75)	0.515
# patients with ≥1 hospital admission	6,212 (26.38%)	6,405 (26.93%)	0.144
# of hospital admissions*	0.43 (0.91)	0.44 (0.96)	0.018
# patients with ≥1 institutional stay	7,267 (30.86%)	7,313 (30.74%)	0.325
# of institutional stays*	1.14 (3.36)	1.10 (3.23)	0.956
# of days hospitalized*	3.01 (9.74)	3.10 (9.27)	0.143
Total # of days hospitalized per arm	70,798	73,837	.

*Continuous variable presented with mean (SD).

Abbreviations: ED=emergency department; mITT=modified intention to treat

© 2022 Pokorney SD et al. *JAMA Network Open.*

eFigure 1. Patient Educational Intervention Mailing Materials

IMPACT-AFib [HEALTH PLAN LOGO]

IMPACT AFib address
IMPACT AFib address

[Date]
[Member Name]
[Member Address]
[Member City, St, zip]

You can lower your risk of stroke.
Bring this letter and pocket card to your next doctor's appointment.
Talk to your doctor about the use of anticoagulant medications to prevent stroke.

Dear [Member Name],

According to our records, you may have been diagnosed with atrial fibrillation. We know that managing your health can be a challenge and hope this information about how to lower your risk for stroke will help.

People who have the heartbeat irregularity known as "atrial fibrillation" are at an increased risk of having a stroke.

*Please visit **IMPACT-AFib.org**, to learn more about atrial fibrillation, stroke risk, and anticoagulant medications. More information about the IMPACT-AFib initiative is available by calling 866-757-0531 or emailing impact-afib@duke.edu*

If you have questions about your benefits, call the number on the back of your health plan ID card.

Talk to your doctor about anticoagulant medications.

This packet contains information about the benefits of taking anticoagulant medications, also called blood thinners, to lower your risk of having a stroke. We recommend that you bring this information packet to your next doctor's appointment. We sent similar information to your doctor.

Anticoagulant medications may not be right for all patients, but they might be right for you. Even if you have talked about this with your doctor in the past, we encourage you to have another conversation about these medications. New anticoagulant medications are safe and effective options for many patients.

"Should I be taking an anticoagulant medication?"

Protecting your health information

We take protecting your health information seriously. None of your health information has been shared with other healthcare organizations. Only you and your doctor were sent this information.

Sincerely,

Chief Medical Officer
Enclosures

If you have any questions, please contact [name] at [phone #] or [email]

Facts about atrial fibrillation, anticoagulant medication, and stroke

- Atrial fibrillation is an abnormal heartbeat in the top chambers of the heart that causes the chambers not to contract (squeeze) normally. This allows blood clots to form in the non-beating chambers.
- Atrial fibrillation increases the risk of a stroke because a blood clot may form in the heart, then travel to the brain causing a stroke.
- Anticoagulants, also known as blood thinners, are a type of medication that reduces the body's ability to form blood clots and decreases the chance of a clot forming in the top chambers of the heart.
- Aspirin is NOT effective in decreasing the risk of stroke.
- Most people with atrial fibrillation should take an anticoagulant medication to reduce their risk of a stroke.

This packet and the packet sent to your doctor are funded by the IMPACT-AFib initiative. This U.S. Food and Drug Administration-sponsored research study is being conducted by [Health Plan], in collaboration with researchers at Harvard Pilgrim Health Care Institute and the Duke Clinical Research Institute. The goal of this initiative is to improve the use of oral anticoagulant medications for stroke prevention in patients with atrial fibrillation.

Disclaimer: Lorem ipsum dolor sit amet, est donec semper pharetra orci, mus ac nec ultricies id, dictum condimentum massa non dapibus. In viteae vestibulum purus facilisis, amet ornare nec quis nec.

© 2022 Pokorney SD et al. *JAMA Network Open.*

IMPACT-AFib

Patient Information

You may have atrial fibrillation and may be at risk of a stroke.

Taking an anticoagulant medication may prevent a stroke.

Atrial fibrillation (AFib) is a heartbeat irregularity. If you have AFib your blood can pool, which increases the risk of a blood clot forming in your heart. The blood clot can travel to your brain, causing a stroke.

Anticoagulant medications, also called blood thinners, can prevent most strokes in patients with AFib. If you are not taking an anticoagulant medication, you may suffer a stroke that could have been prevented.

Please review this information and talk with your doctor to find out if you should be on an anticoagulant medication to prevent a stroke.

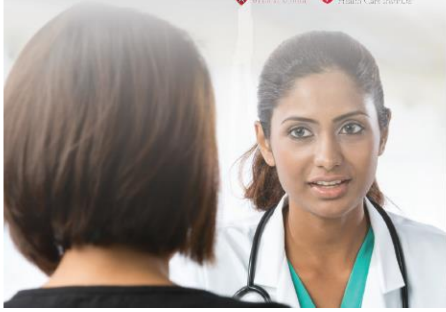

How do I know if I'm at a high risk for stroke?
If you have AFib, you are at a higher risk of stroke. You are at additional risk if you:

- Have high blood pressure
- Have high blood sugar
- Have weak heart function
- Have had a stroke or mini-stroke
- Have had a heart attack or a blocked vessel in your leg
- Are over 64 years old
- Are a woman

I have AFib only sometimes. Am I still at risk for a stroke?

Yes, the risk is similar whether your AFib is all the time, often, or only occasionally.

What is an anticoagulant?
Anticoagulants are medications that:

- Prevent blood clots
- Keep existing clots from moving

Examples include: Coumadin®, Eliquis®, Pradaxa®, Savaysa®, warfarin, and Xarelto®.*

**The information in this mailing is NOT sponsored by any drug company.*

For more information, please visit IMPACT-AFib.org

If my doctor prescribes an anticoagulant, how should I take it?

- Take your medication exactly as directed by your doctor
- Take it at the same time each day
- If you forget to take your medication one day, take a dose as soon as possible on the same day
- Do not take a double dose the following day to "catch up"

Tell your doctor if you are pregnant or plan to become pregnant, are breastfeeding or plan to breastfeed, if you have liver or kidney problems, or are planning to have surgery.

Will anticoagulant medications prevent strokes?

- Anticoagulant medications reduce the risk of stroke by 70% in patients with atrial fibrillation.

What about aspirin?

- Aspirin is **not** an effective medication for decreasing the risk of stroke caused by atrial fibrillation.

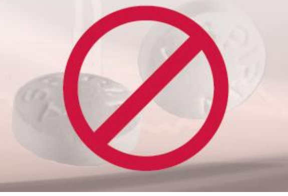

How can I keep myself safe from bleeding and falls?
(As with other medications, there is a risk of experiencing side effects while taking anticoagulants. The main side effect is that you can bleed too easily.)

- Use a soft bristle toothbrush and waxed dental floss
- Use an electric razor to shave
- Be careful with sharp objects: toothpicks, knives, tools, scissors, etc.
- Wear shoes or non-skid slippers at all times
- Avoid nonsteroidal anti-inflammatory drugs like ibuprofen, naproxen, etc.
- Be careful when trimming toenails or callouses
- Avoid activities that increase risk of falls or involve hard contact, such as contact sports

Is it OK to take an anticoagulant medication if I have had bleeding? What if I fall?

- If you are at high risk for bleeding, the use of an anticoagulant medication depends on whether the benefit of preventing a stroke is more important than the risk of bleeding. Talk with your doctor about your risk.
- The benefits of preventing stroke outweigh the risk of bleeding for many people who might fall.

If I have bleeding, is there something to reverse the effect of anticoagulant medications? An antidote?

- Yes, there are antidotes for warfarin and Pradaxa®
- Reversal drugs are in development for other anticoagulant medications
- There is no antidote for aspirin

Will an anticoagulant medicine interact with other medicines or foods?

- Warfarin interacts with foods that are high in vitamin K
  - You should ask your doctor or pharmacist for a list of food interactions
- Xarelto® should be taken with food to help your body absorb the medicine

Talk with your doctor or pharmacist if you have questions about any medications or foods that might affect your anticoagulant medication, including nonprescription medicines, vitamins, and herbal supplements.

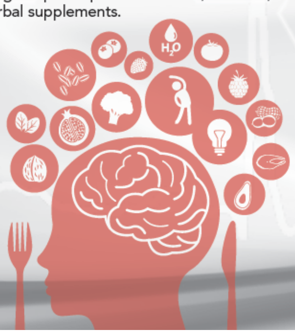

Am I at risk for stroke?

- The CHA$_2$DS$_2$-VASc calculates stroke risk for patients with atrial fibrillation.
- Complete the following CHA$_2$DS$_2$-VASc calculator to determine your personal risk.
- If you have AFib and a CHA$_2$DS$_2$-VASc score of 2 or greater, you have an increased risk of stroke.

CHA$_2$DS$_2$-VASc RISK SCORE	If yes, add points
Do you have congestive heart failure?	+1
Do you have high blood pressure or are you taking blood pressure medication(s)?	+1
Are you between 65–74 years of age?	+1
Are you 75 years old or older?	+2
Do you have diabetes?	+1
Have you ever had a stroke or TIA (mini-stroke)	+2
Have you ever had vascular disease (bypass surgery, heart attack, peripheral artery disease, or aortic plaque)?	+1
Are you female?	+1
MY TOTAL	

© 2022 Pokorney SD et al. *JAMA Network Open.*

IMPACT-AFib

Dear Doctor,
As you know, patients who have atrial fibrillation and a CHA_2D_S2-VASc score of 2 or more are at risk of a stroke and would likely benefit from taking an anticoagulant medication.

The CHA_2DS_2-VASc calculator shows a patient's risk of a stroke. The score helps determine whether a patient would benefit from an anticoagulant.

CHA_2DS_2-VASC CALCULATOR

	Risk factor	If patient has risk factor, add points
C	Congestive Heart Failure	+1
H	High Blood Pressure (hypertension, including normal blood pressure on blood pressure medications)	+1
A_2	Age 75 years old or older	+2
D	Diabetes	+1
S_2	Stroke or TIA (mini-stroke)	+2
V	Vascular Disease (prior bypass surgery, heart attack peripheral artery disease, or aortic plaque)	+1
A	Age 65-74 years	+1
Sc	Sex Category: Female sex	+1
	**TOTAL**	

Patients with a score of 2 or greater are at high risk for stroke. Strokes may be prevented with an anticoagulant medication.

Is IMPACT-AFib new to you? For more information, please visit **IMPACT-AFib.org.**

Duke Clinical Research Institute

DEPARTMENT OF POPULATION MEDICINE
HARVARD MEDICAL SCHOOL
Harvard Pilgrim Health Care Institute

FDA U.S. FOOD & DRUG ADMINISTRATION

© 2022 Pokorney SD et al. *JAMA Network Open.*

IMPACT-AFib

Dear Patient,

Talk with your doctor to find out if taking an anticoagulant medication is right for you. Anticoagulant medications prevent blood clots and are sometimes called blood thinners.

Not sure where to start? Here is a way to start the conversation with your doctor:
I received this letter, information sheet, and card in the mail from my health plan. My health plan suggests that I talk with my physician about taking an anticoagulant to help prevent a stroke.

QUESTIONS TO ASK YOUR DOCTOR:	
**1.**	Do I have atrial fibrillation?
**2.**	What are my risk factors for stroke?
**3.**	Based on my CHA_2DS_2-VASc score from the other side of this card, should I be taking an anticoagulant medication?<br>• If no, why not?<br>• If yes, what kind of anticoagulant would work best for me?
**4.**	Is aspirin good for me to take? The information I received said it was not effective in preventing stroke caused by AFib.
**5.**	Should I be concerned with bleeding if I take an anticoagulant?

Most anticoagulant medications are covered by your health plan. Check with your health plan's information or call the phone number on your health plan card to determine which of the following drugs are covered: Coumadin®, Eliquis®, Pradaxa®, Savaysa®, warfarin, and Xarelto®.*

For additional information, please visit **IMPACT-AFib.org.**

**This mailing is NOT sponsored by any drug company.*

Duke Clinical Research Institute

DEPARTMENT OF POPULATION MEDICINE
HARVARD MEDICAL SCHOOL
Harvard Pilgrim Health Care Institute

© 2022 Pokorney SD et al. *JAMA Network Open.*

eFigure 2. Provider Educational Intervention Mailing Materials

U.S. FOOD & DRUG ADMINISTRATION

IMPACT-AFib

Duke Clinical Research Institute

DEPARTMENT OF POPULATION MEDICINE HARVARD MEDICAL SCHOOL Harvard Pilgrim Health Care Institute

[HEALTH PLAN LOGO]

Dear Provider:

As part of our effort to improve the use of oral anticoagulant medications for stroke prevention in patients with atrial fibrillation (AFib), we would like to introduce you to the IMPACT-AFib initiative. The objective of the IMPACT-AFib initiative is to increase awareness and education among you and your patients. This FDA-sponsored initiative is being conducted by [HEALTH PLAN] in collaboration with researchers at Harvard and Duke.

Educational materials were sent to patient(s) who appear to have AFib, have high stroke risk (CHA_2DS_2-VASc score ≥ 2), and have no record available to us of having filled a prescription for an anticoagulant in the past year. Please see the following page for a list of patients who received these materials.

Facts about AFib

- Patients with AFib have a five times higher stroke risk relative to patients without AFib *(Circulation 2011;123(10):e269–367)*
- More than two-thirds of strokes caused by AFib are preventable with anticoagulation *(Annals of internal medicine 146.12 (2007): 857–867)*
- 50 percent of patients with AFib and high stroke risk have not filled an anticoagulant prescription *(Circulation 2014; 129 (15),1568–1576)*

Common misperceptions about stroke prevention	
**Aspirin is good enough**	• Aspirin reduces stroke by < 20%, if at all, compared with 70% reduction with anticoagulation; therefore, aspirin is not sufficiently effective for stroke prevention[1]
**Patients with AFib are at greater risk of bleeding than stroke**	• 30% of elderly patients fall in a year, but a patient would need to fall nearly every day before the risk of intracranial bleeding outweighs the benefits of anticoagulants.[2]<br>• The risk of recurrent GI bleeding averages 1.2% per year but would have to exceed 10% before the risk of GI bleeding outweighs the benefit of anticoagulants.[3]

There are appropriate reasons for patients to not take an anticoagulant, including pregnancy and history of intracranial hemorrhage. A response mailer is enclosed for you to share these reasons, should they exist for your patient(s).

[1] European Heart Journal 2015; 36: 653-656 [2] *Arch Intern Med 1999;159:677-685* [3] *Arch Intern Med 2002;162:541-550*

U.S. FOOD & DRUG ADMINISTRATION

IMPACT-AFib

Duke Clinical Research Institute

DEPARTMENT OF POPULATION MEDICINE HARVARD MEDICAL SCHOOL Harvard Pilgrim Health Care Institute

[HEALTH PLAN LOGO]

What should you do?

Please review and discuss anticoagulation and stroke risk with your patient(s) at their next visit.

For Health Plan pharmacy coverage policy, go to www.HealthPlan.com. You can visit IMPACT-AFib.org or call 866-757-0531, or email impact-afib@duke.edu for more information about this initiative.

If you have questions or concerns, please contact us at [MD@HealthPlan.com] or [xxx-xxx-xxxx].

Enclosed is an information card and the patient information.

Sincerely,

Chief Medical Officer
Health Plan

Name	Date of Birth

This packet and the packet sent to your patient(s) are funded by the IMPACT-AFib initiative. This U.S. Food and Drug Administration-sponsored research study is being conducted by [Health Plan], in collaboration with researchers at Harvard Pilgrim Health Care Institute and the Duke Clinical Research Institute. The goal of this initiative is to improve the use of oral anticoagulant medications for stroke prevention in patients with atrial fibrillation.

Disclaimer: Lorem ipsum dolor sit amet, est donec semper pharetra orci, mus ac nec ultricies id, dictum condimentum massa non dapibus. In viteae vestibulum purus facilisis, amet ornare nec quis nec.

© 2022 Pokorney SD et al. *JAMA Network Open.*

IMPACT-AFib	IMPACT-AFib

Patients with atrial fibrillation (AFib) are at five times higher risk of stroke *(Circulation 2011;123(10):e269–367)*

- Two-thirds of strokes in patients with atrial fibrillation are preventable with anticoagulation, as recommended in clinical practice guidelines *(Annals of internal medicine 146.12 (2007): 857–867)*
- Despite this guideline, at least 50% of patients with a CHA_2DS_2-VASc score of 2 or higher are not being prescribed an oral anticoagulant *(Circulation 2014; 129 (15), 1568–1576)*

You can help change these statistics by—

- Educating all of your patients with AFib about anticoagulant use
- Stopping the use of aspirin as an anticoagulant

For more information, please visit IMPACT-AFib.org

If they are not taking oral anticoagulants, patients with AFib can have strokes that are PREVENTABLE.

Myth vs. Reality
**Myth:** Aspirin prevents stroke and is safe
**Reality:** Aspirin is neither safe nor effective *(Eur Heart J 2015;36:653-6)*
**Myth:** It is risky to resume oral anticoagulation therapy in the months after bleeding
**Reality:** Benefits generally outweigh risks *(Arch Intern Med 2002;162:541-550)*
**Myth:** It is risky to prescribe oral anticoagulants to patients who are at risk of falling
**Reality:** "… persons taking warfarin must fall about 295 times in 1 year for warfarin to not be the optimal therapy." *(Arch Intern Med 1999;159:677-685)*
**Myth:** Patients who don't tolerate warfarin won't tolerate any oral anticoagulant
**Reality:** Most patients tolerate novel oral anticoagulants *(N Engl J Med 2011;364:806-17)*
**Myth:** Patients with paroxysmal AFib are low risk of stroke
**Reality:** Risk is about the same for paroxysmal or permanent AFib, indicating need for anticoagulation *(Circulation 2014;130: e199-e267)*
**Myth:** There is no antidote for novel oral anticoagulants
**Reality:** An injectable reversal agent (Praxbind) is available for the novel oral anticoagulant Pradaxa (dabigatran). *(N Engl J Med. 2015;373:511-20)*

© 2022 Pokorney SD et al. *JAMA Network Open.*

Duke Clinical Research Institute

DEPARTMENT OF POPULATION MEDICINE
HARVARD MEDICAL SCHOOL
Harvard Pilgrim Health Care Institute

If we have incorrectly identified a patient as being
able to benefit from taking an oral anticoagulant,
we would like to hear from you.

Please complete the following information, then
seal and return this mailer.

IMPACT-AFib

Patient name: ____

Date of birth: _____ /_____ / 19____
Month Day Year

Should not be prescribed an oral anticoagulant because— *(please check all that apply)*

- ☐ He/she is not my patient and/or I am not the prescribing physician
- ☐ Patient does not have atrial fibrillation
- ☐ Patient already takes an anticoagulant
- ☐ An anticoagulant has already been prescribed
- ☐ Very high risk of major/life-threatening bleeding
- ☐ Unable to tolerate warfarin
- ☐ Unable to afford a non-vitamin K oral anticoagulant
- ☐ Patient decision after thorough review of risks, benefits, concerns
- ☐ Other (please explain): ______________________________________

© 2022 Pokorney SD et al. *JAMA Network Open.*

eFigure 3. Sensitivity Analysis of Primary Endpoint Results. A Forest plot that shows the primary endpoint of OAC initiation at 1 year, at 183 days, at 90 days, and at 42 days with modified intention to treat for the sensitivity analysis of 5 data partners.

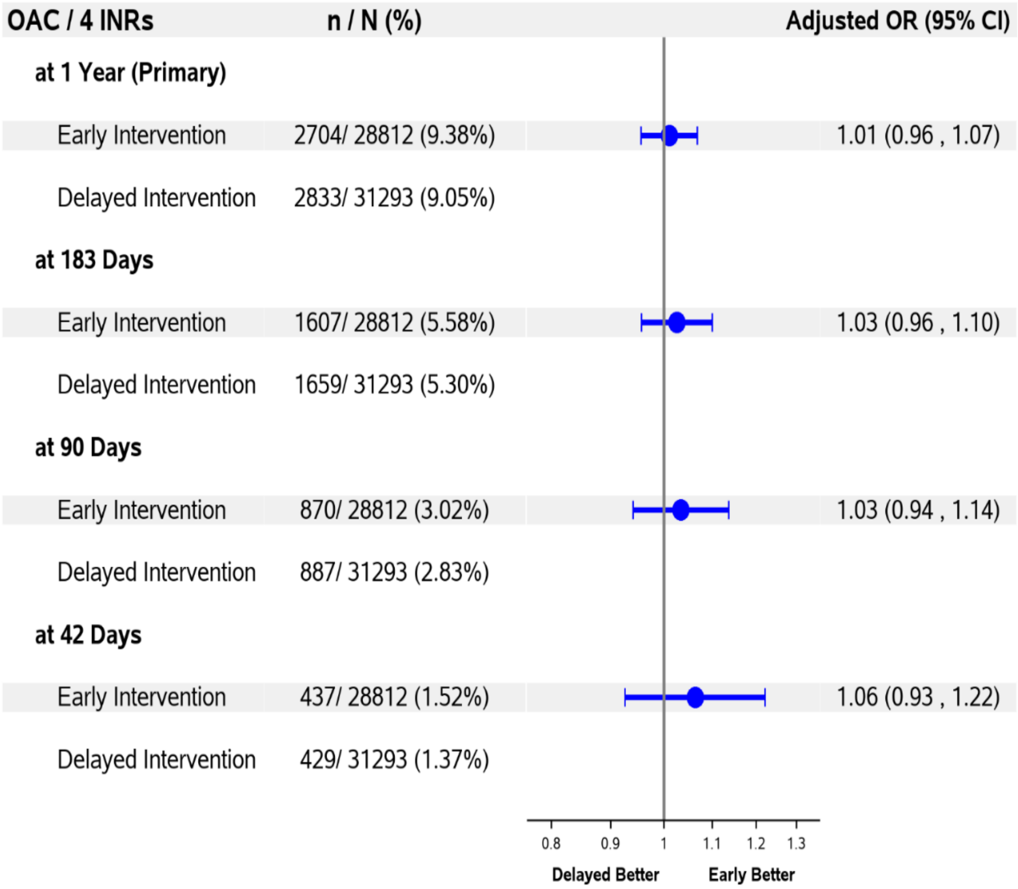

© 2022 Pokorney SD et al. *JAMA Network Open.*

eFigure 4. Sensitivity Analysis of Primary Endpoint Subgroups. A Forest plot that shows the primary outcome of OAC initiation at 1 year with modified intention to treat across pre-specified subgroups for the modified intention to treat for the sensitivity analysis of 5 data partners.

Subgroup	Early Intervention n / N (%)	Delayed Intervention n / N (%)	Adjusted OR (95% CI)
**Sex**			
Female	1350 / 14031 (9.62%)	1343 / 15050 (8.92%)	1.05 (0.97,1.14)
Male	1354 / 14781 (9.16%)	1490 / 16243 (9.17%)	0.97 (0.90,1.05)
**Age***			
< 65	152 / 1579 (9.63%)	160 / 1671 (9.58%)	1.00 (0.79,1.26)
65 - 74	774 / 7697 (10.06%)	811 / 8209 (9.88%)	1.00 (0.90,1.12)
75 - 84	1177 / 11256 (10.46%)	1202 / 12137 (9.90%)	1.03 (0.95,1.12)
>= 85	529 / 7419 (7.13%)	576 / 8437 (6.83%)	1.01 (0.89,1.14)
**CHA2DS2-VASC Score**			
2 - 3	728 / 8184 (8.90%)	798 / 8652 (9.22%)	0.95 (0.86,1.06)
4 - 5	1210 / 12415 (9.75%)	1225 / 13663 (8.97%)	1.07 (0.98,1.16)
>=6	764 / 8197 (9.32%)	809 / 8953 (9.04%)	0.99 (0.89,1.09)

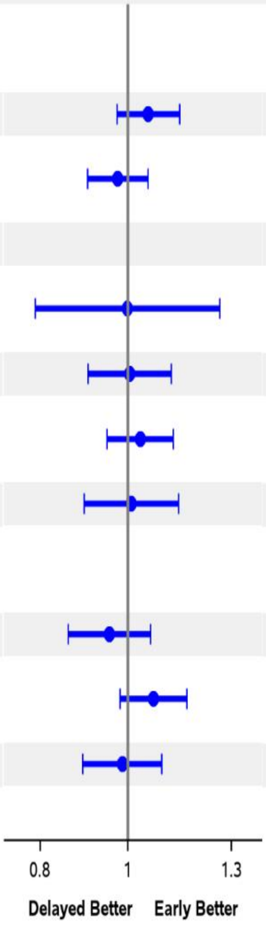

*meta-analysis were conducted using data from 4 sites.

© 2022 Pokorney SD et al. *JAMA Network Open.*

eFigure 5. Sensitivity Analysis of Secondary Clinical Outcomes. A Forest plot that shows secondary clinical outcomes of at 1 year with the modified intention to treat population from the sensitivity analysis of 5 data partners.

	Early Intervention Cumulative Incidence % (95% CI)	Delayed Intervention Cumulative Incidence % (95% CI)	Adjusted HR (95% CI)
**Hospitalization for**			
ischemic/ unknown stroke*	1.65% (1.49%, 1.82%)	1.80% (1.64%, 1.96%)	0.88 (0.77,1.01)
hemorrhagic stroke**	0.41% (0.32%, 0.49%)	0.36% (0.28%, 0.43%)	1.17 (0.87,1.57)
ischemic/ hemorrhagic stroke*	2.03% (1.85%, 2.21%)	2.18% (2.00%, 2.36%)	0.92 (0.81,1.05)
ischemic/ hemorrhagic stroke / systemic embolism*	2.12% (1.94%, 2.31%)	2.28% (2.10%, 2.47%)	0.94 (0.83,1.06)
any bleeding event*	2.10% (1.92%, 2.29%)	2.16% (1.98%, 2.34%)	1.01 (0.90,1.14)
ischemic/ hemorrhagic stroke/ systemic embolism/ bleeding*	3.75% (3.51%, 3.99%)	3.86% (3.62%, 4.10%)	0.96 (0.88,1.05)
**All-cause in-hospital death***	2.46% (2.26%, 2.66%)	2.40% (2.22%, 2.59%)	1.01 (0.91,1.13)

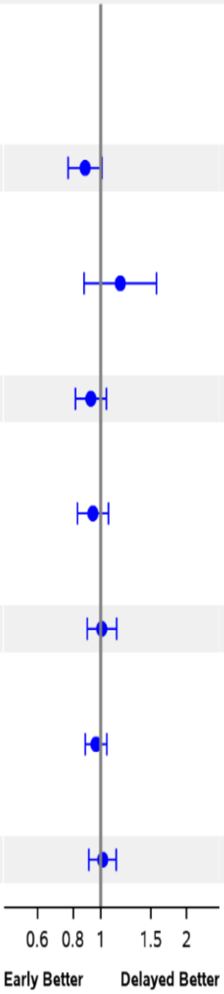

*meta-analysis were conducted using data from 4 sites.

© 2022 Pokorney SD et al. *JAMA Network Open.*
