# Supplementary material for: Effect of Mailing Educational Material to Patients With Atrial Fibrillation and Their Clinicians on Use of Oral Anticoagulants: A Randomized Clinical Trial
Source: JAMA Netw Open. 2022 May 31;5(5):e2214321. doi: 10.1001/jamanetworkopen.2022.14321 (PMC9157265; doi:10.1001/jamanetworkopen.2022.14321)
Supplement: Supplement 3. — Data Sharing Statement [file jamanetwopen-e2214321-s00.pdf]

Data Sharing Statement

Pokorney. Effect of Mailing Educational Material to Patients With Atrial Fibrillation and Their Clinicians on Use of Oral Anticoagulants. *JAMA Netw Open*. Published May 31, 2022. doi:10.1001/jamanetworkopen.2022.14321

Data
Data available: No
